# Plasma extracellular vesicles reflect response and prognosis in patients with breast cancer undergoing neoadjuvant treatment

**DOI:** 10.1186/s13058-025-02209-0

**Published:** 2026-01-09

**Authors:** Karin Ekström, Nazia Riaz, Karolina Larsson, Afrodite Nemeth, Rossella Crescitelli, Barbro Linderholm, Roger Olofsson Bagge

**Affiliations:** 1 Department of Surgery, Institute of Clinical Sciences, Sahlgrenska Academy, University of Gothenburg, Gothenburg, Sweden; 2 Department of Oncology, Institute of Clinical Sciences, Sahlgrenska Academy, University of Gothenburg, Gothenburg, Sweden; 3 Department of Oncology, Sahlgrenska University Hospital, Gothenburg, Sweden; 4 Faculty of Information Technology and Bionics, Pázmány Péter Catholic University, Budapest, Hungary; 5 Department of Surgery, Sahlgrenska University Hospital, Gothenburg, Sweden

**Keywords:** Extracellular vesicles, Exosomes, Breast cancer, Neoadjuvant systemic treatment, Biomarkers, Liquid biopsy

## Abstract

**Background:**

Extracellular vesicles (EVs) are emerging as non-invasive biomarkers in cancer, but their role in monitoring the response to neoadjuvant systemic treatment (NST) in patients with breast cancer remains unclear. This study aimed to assess whether EV concentration and surface marker profiles in plasma reflect treatment response and clinical outcome in patients with early-stage breast cancer receiving NST.

**Methods:**

Plasma samples were collected from 59 patients with luminal B-like, HER2-positive, or triple-negative breast cancer before and after NST. EVs were isolated by size exclusion chromatography and characterized by nanoparticle tracking analysis, electron microscopy, Western blotting, and MACSPlex surface marker profiling. Paired samples were available for 29 patients, allowing longitudinal analysis.

**Results:**

Patients who achieved pathological complete response (pCR) had significantly lower baseline EV concentrations than those with residual disease. Post-treatment EV levels were also lower in patients who remained free from distant metastasis and had improved breast cancer-specific survival. EV surface marker profiling revealed that CD69, CD29, and CD49e were reduced after NST, whereas CD44 was increased. Notably, EpCAM levels increased specifically in non-PCR patients, suggesting persistent tumor-derived EV release, whereas SSEA-4 levels increased only in patients who achieved pCR. Although EV concentrations and markers differed by subtype and outcome, changes in EV levels following NST were not independently predictive of prognosis.

**Conclusions:**

These findings support the potential of plasma-derived EVs as dynamic biomarkers of treatment response, and suggest possible prognostic relevance in breast cancer. Validation in larger, independent cohorts is needed to assess their clinical applicability.

**Supplementary Information:**

The online version contains supplementary material available at 10.1186/s13058-025-02209-0.

## Background

Breast cancer is the most commonly diagnosed cancer worldwide, with approximately 2.3 million new cases each year, and it remains the leading cause of cancer death among females [1]. Treatments include systemic oncological therapy, surgery, and radiotherapy. Treatment recommendations are based on tumor stage, breast cancer subtype, and patient-related factors [2]. The breast cancer subtype is key to determining prognosis and to tailoring treatment and is based on the expression of tumor cell biomarkers [3]. The primary biomarkers used for subtype classification include expression of estrogen receptor (ER), progesterone receptor, and human epidermal growth factor receptor 2 (HER2). On the bases of these markers and the cancer cell proliferation, breast cancers are commonly categorized into four main subtypes: luminal A (ER + , HER2-, low grade, low proliferative index) luminal B (ER + , HER2-, high grade, high proliferative index), HER2-positive (ER ± , HER overexpressed or amplified) and triple-negative breast cancer (TNBC) (ER-, PR-, HER2-) [4].

The prognosis of patients with breast cancer has improved over time, with a 5-year survival rate of 90% in countries with access to early diagnostics and modern care [5]. However, there remains an unmet need to further improve outcomes for patients with poor prognoses while avoiding overdiagnosis and overtreatment. In patients with a more advanced ER + /HER2- breast cancer (stage III), which typically corresponds to the luminal B-like subtype, or in patients with early-stage (stage I-II) HER2 + or TNBC, neoadjuvant systemic treatments (NSTs) including chemotherapy, targeted therapies, and immunotherapy are recommended [2]. It offers the opportunity to increase the rates of breast-conserving surgery and provides prognostic information where patients with a pathologic complete response (pCR) have favorable outcomes [6]. Consequently, response-guided treatment allows for postoperative escalation of therapies in patients who do not achieve pCR, leading to improved outcomes [7, 8]. This underscores the importance of investigating novel biomarkers that may improve the understanding of the underlying biological mechanisms of tumor response to therapies and to enable real-time monitoring during NST, ultimately supporting more precise treatment recommendations.

Extracellular vesicles (EVs) are a diverse group of non-replicative, lipid bilayer-enclosed particles that are released into the extracellular environment by various cell types through an evolutionarily conserved process [9–12]. Under physiological conditions, the packaged EV cargo, including but not limited to bioactive proteins, lipids, and nucleic acids, facilitates intercellular communication, maintains homeostasis, and supports immune surveillance [11, 12]. Given that biological processes are highly dependent upon the cell of origin and molecular content of the EVs, the specific composition of their cargo shapes both their fate and function, thereby underscoring the essential role of cargo-sorting mechanisms during EV biogenesis [12, 13].

In line with these physiological roles, emerging evidence highlights EVs as key mediators of tumorigenesis and the formation of a premetastatic niche in both hematological and solid organ malignancies, including breast cancer [14, 15]. Importantly, the oncogenic cargo derived from both the cancer and the stromal cells has been linked with oncogenesis through complex molecular mechanisms [16, 17]. In the context of breast cancer, preclinical studies have shown that hypoxia triggers increased EV production from breast cancer cells, leading to expansion of the stem cell pool, epithelial-mesenchymal transition, and S100A9-mediated immunosuppression [18, 19]. Moreover, EVs have also been implicated in therapy resistance by reactivating the self-renewal capacity of dormant, therapy-resistant cells in hormone receptor-positive breast cancer [20].

There is a growing interest in the evaluation of breast cancer-derived EVs as potential liquid biopsy-based biomarkers [21, 22]. Although this work is in progress, several advantages are attributed to EVs as potential liquid biopsy-based biomarkers, including high biological stability, their presence in almost all body fluids, and potential for analysing multiple targets contained in the EV cargo. Additionally, because EVs are released by viable cells, they provide a more relevant snapshot of the cellular state, offering greater representativeness than biomarkers such as cell-free DNA, which may also be derived from dying or apoptotic cells [23, 24].

To date, however, few studies have analyzed matched pre- and post-treatment EV profiles in early-stage breast cancer patients receiving NST specifically in the context of pCR [25]. It remains to be determined whether the dynamics of EV quantification vary with NST and whether they can be used as biomarkers for response assessment to NST. In this study, we aimed to assess whether EV concentrations and surface marker profiles differ by breast cancer subtype and response to NST in pre- and post-treatment plasma samples. Moreover, we investigated whether EV features were associated with clinical outcomes.

## Materials and methods

### Patient cohort and sample collection

This prospective, translational study was conducted at Sahlgrenska University Hospital (Gothenburg, Sweden) to evaluate whether plasma extracellular vesicle (EV) concentration and surface marker profiles reflect treatment response and prognosis in patients with early-stage breast cancer undergoing neoadjuvant systemic treatment (NST). Informed consent was obtained from each participant in accordance with the Declaration of Helsinki and with the principles of good clinical practice. The study protocol was approved by the regional ethical review board in Gothenburg (Dnr 221–17). Chemotherapy regimens primarily consisted of anthracyclines and taxanes. Those with HER2-positive disease additionally received HER2-targeted monoclonal antibodies, including trastuzumab and pertuzumab. Patients with TNBC were treated with chemotherapy alone. As the study period preceded the clinical implementation of immunotherapy, no patients received immune checkpoint inhibitors in the NST setting. Surgical treatment consisted of either mastectomy or breast-conserving surgery, along with axillary surgery. All patients were managed according to standard clinical routines in line with Swedish National Guidelines for breast cancer [26]. Postoperative radiotherapy was administered when clinically indicated. For patients with luminal breast cancer, endocrine therapy was provided postoperatively. The study cohort is representative of high-risk breast cancer patients receiving NST, with a wide age range and clinical diversity. Nearly all patients scheduled for NST during the inclusion period were approached for participation. We have submitted all relevant data of our experiments to the EV-TRACK knowledgebase (EV-TRACK ID: EV250083) (Van Deun J, et al. EV-TRACK: transparent reporting and centralizing knowledge in extracellular vesicle research. Nature methods. 2017;14(3):228–32).

Peripheral blood samples were collected from 59 patients with early-stage breast cancer in EDTA tubes at two time points: before the initiation of NST, (58 patients) and after the completion of NST but prior to surgery (31 patients). In total, 29 patients had paired plasma samples available from both time points. Blood samples were centrifuged at 2,000 × *g* for 10 min at room temperature to separate the plasma. The plasma was carefully aliquoted into sterile cryotubes and stored at − 80 °C until EV isolation.

### Isolation of extracellular vesicles

EVs were isolated from human plasma via size exclusion chromatography (SEC) with qEV1 70 nm columns (IZON Science, New Zealand) in combination with an automatic fraction collector (AFC, IZON Science), following the manufacturer’s protocol with minor optimizations. Briefly, 1.1 mL of plasma was centrifuged at 3,000 × g for 10 min at 4 °C. After centrifugation, 1.0 mL of the resulting supernatant was directly applied to the pre-equilibrated qEV1 columns, which were mounted on the AFC. EV isolation was performed via the default AFC program, which automatically collects a 2.8 mL EV-enriched fraction following a void volume of 4.7 mL, according to the manufacturer’s specifications. The EV-containing fractions were immediately placed on ice after collection. Between each run, the columns were flushed with 13.5 mL of 0.5 M NaOH followed by 27 mL of PBS. The columns were reused for up to five runs on the same day but were not stored or reused on subsequent days.

The collected EV fractions were concentrated by ultrafiltration using Amicon Ultra-4 centrifugal filter units with a 100 kDa molecular weight cutoff (Millipore). The filters were pre-wetted with phosphate buffered saline (PBS) and centrifuged at 3,200 × g for two minutes before the sample was added. EV samples were then centrifuged at 3,200 × g for 20 min at 10 °C. The final volume was verified, and if it exceeded 100 µL, further centrifugation was performed until the volume was reduced to less than 100 µL. The concentrate was then adjusted to a final volume of 100 µL with PBS. EV aliquots were stored at -80 °C until downstream analysis. All EV samples were used for nanoparticle tracking analysis (NTA), Qubit, and MACSPlex analysis. TEM (n = 2) and western blotting (*n* = 3, matched pre- and post NST EV samples) were performed on selected samples for EV characterization.

### Nanoparticle tracking analysis

Nanoparticle tracking analysis (NTA) was performed via the ZetaView® PMX120 system (Particle Metrix, Germany) to determine the concentration and size distribution of the EVs. The instrument was calibrated with 100-nm polystyrene alignment beads (Sigma). For each sample, three independent dilutions were prepared in PBS (technical triplicates), with dilution factors typically ranging from 1:10,000 to 1:40,000 depending on the sample concentration. Each dilution was introduced into the instrument port via a syringe, and measurements were acquired at eleven distinct cell positions, with three cycles recorded per position. The concentration and size of the EVs were analyzed via ZetaView® software (version 8.05.11, SP1), with the camera sensitivity set to 80 and the shutter speed set to 100. The brightness threshold was set to 20, and the particle size detection range was defined between 10 and 1000 nm.

### Transmission electron microscopy

The investigation of EVs by negative staining was performed as previously described [27]. Briefly, 5 μg of vesicles was placed onto glow-discharged 200-mesh Formvar/carbon copper grids (Electron Microscopy Sciences, USA). After two washes in H_2_O, EVs were fixed in 2.5% glutaraldehyde and washed twice in H_2_O. The samples were then stained with 2% uranyl acetate for 1.5 min. Negative-stained samples were investigated using a Talos L120C electron microscope (Thermo Fisher Scientific, USA) at 120 kV with a CCD camera.

### Qubit protein assay

The protein concentration of the EV samples was determined using the Qubit™ Protein Assay Kit with a Qubit™ 3 Fluorometer (ThermoFisher Scientific), following the manufacturer’s protocol. Briefly, 1 µL of each EV sample was mixed with 199 µL of working solution and incubated for 15 min at room temperature in dark. The fluorescence was then measured. Samples exceeding the standard curve range were further diluted to adjust the concentration within the range of the assay. All measurements, including those of standards and EV samples, were performed in technical triplicate.

### Western blot analysis

Western blot analysis was performed on three pre- and post-treatment samples (pre-luminal B, pre-HER2, pre-TNBC, post-luminal B, post-HER2, post-TNBC) for protein markers as specified in the MISEV guidelines [9]. As positive controls, cell lysates were used for the detection of CD9, Flotillin-1, and Calnexin, whereas a plasma sample was used as control for ApoA1. The protocol has been described previously [28, 29]. Briefly, 10 or 20 μg of protein was loaded into the wells of precast 4–20% polyacrylamide Mini-PROTEN TGX gels (Bio-Rad Laboratories, Hercules, CA, USA). SDS-PAGE gel electrophoresis was performed by running the gel at 80 V for 5 min and then at 180 V for approximately one hour. The gels were then placed on the ChemiDoc MP Imaging system (Bio-Rad Laboratories) for assessment of electrophoresis before transfer and activated by exposure to UV light (set at autoexposure). Proteins were then transferred to polyvinylidene fluoride (PVDF) membranes (Bio-Rad Laboratories) using the Trans-Blot Turbo system (Bio-Rad Laboratories), followed by blocking for 1 h in Every Blot Blocking Buffer (Bio-Rad Laboratories). This was followed by overnight incubation with the following dilutions of the primary antibodies at 4 °C: anti-Flotillin (1:1000, clone EPR6041, Abcam), anti-Calnexin (1:1000, clone C5C9, Cell Signaling Technology), anti-CD9 (1:1000, clone MM2/57, Millipore) and anti-ApoA1 (1:500, GTX112692, GeneTex). Non-reducing conditions were used for running the gel for CD9. The membranes were rinsed three times in 1X Tris-buffered saline-Tween (TBST) and incubated with the appropriate secondary antibodies (anti-rabbit, Amersham ECL rabbit IgG, HRP linked NA934OV, 1:5000 and anti-mouse Amersham ECL mouse IgG, HRP linked, NA9310, 1:1000) and Precision Protein StrepTactin-HRP Conjugate (Bio-Rad Laboratories) for 1 h. All antibody dilutions were performed in Every Blot Blocking Buffer (Bio-Rad Laboratories). The membranes were rinsed three times in 1 X TBST and analyzed with SuperSignal West Femto maximum sensitivity substrate (Thermo Fisher Scientific) on a ChemiDoc Imaging System (Bio-Rad Laboratories.

### Bead-based multiplex analyses of extracellular vesicles

Surface protein profiling of EVs was performed using the MACSPlex EV kit IO (Miltenyi Biotec, Germany) following the manufacturer’s overnight protocol for the MACSPlex Filter Plate with minor modifications. The kit enables simultaneous detection of 37 surface markers (CD1c, CD2, CD3, CD4, CD8, CD9, CD11c, CD14, CD19, CD20, CD24, CD25, CD29, CD31, CD40, CD41b, CD42a, CD44, CD45, CD49e, CD56, CD62p, CD63, CD69, CD81, CD86, CD105, CD133.1, CD142, CD146, CD209, CD326, HLA-ABC, HLA-DR DP DQ, MCSP, ROR1 and SSEA-4) and includes two isotype controls (mIgG1 and REA control) corresponding to the antibodies. Each patient sample was analyzed in technical triplicates, and three blanks (PBS in MACSPlex buffer) were included in parallel. A total of 1 × 10^10^ EVs per sample were added to each well to a final volume of 120 µl. Subsequently, 15 µl of MACSPlex capture beads were added to each well, and the plate was incubated overnight on an orbital shaker at room temperature in the dark. After incubation, the beads were washed with buffer, and each wash step was followed by centrifugation at 300 × g for three minutes at room temperature. Following the washes, 15 µl of the detection cocktail (5 µl each of CD9, CD63, and CD81) was added to each well to a final volume of 150 µl. The samples were incubated for one hour on an orbital shaker at room temperature in the dark. After incubation, the beads were washed twice, resuspended in 200 µl of buffer, and transferred to flow cytometry tubes. Samples were acquired on a BD FACSVerse™ flow cytometer via BD FACSuite™ software (BD Biosciences, USA), with fluorescence for FITC, PE, and APC recorded. A total of 10,000 beads per sample were analyzed.

The bead populations were gated based on PE and FITC fluorescence using FlowJo Software (v10.9), and median fluorescence intensity (MFI) values for APC were exported. Background-subtracted MFI values were calculated by subtracting the corresponding isotype controls (REA or mIgG1). These values were then normalized to the mean APC MFI of CD9, CD63, and CD81. The negative values were set to zero. A detection filter was applied by retaining only markers with a normalized MFI above 0.5 in at least 50% of the samples. For these markers, values below the 0.5 threshold were subsequently set to 0.5 before statistical analyses. All filtering and thresholding steps were performed in Qlucore Omics Explorer (Qlucore AB, Sweden), and markers that did not fulfill these criteria were excluded from further analysis.

### Statistical analysis

One-way ANOVA was used to compare breast cancer subtypes, and unpaired two-tailed t-tests were applied for two-group comparisons (e.g., pCR vs. non-pCR). Paired 2-tailed t-tests were used to assess differences between pre- and post-treatment samples. Pearson correlation analysis was used to evaluate associations between EV concentration and continuous clinical variables. Survival analyses were performed using the Kaplan–Meier method, and differences between groups were assessed using the log-rank test, with EV concentration dichotomized at the median. Breast cancer-specific survival (BCSS) was defined as the time from diagnosis to death specifically attributed to breast cancer. Distant metastasis-free survival (DMFS) was defined as the time from the first blood sample to either distant metastasis or death, whichever occurred first. Both endpoints were assessed using a landmark analysis at four years. For the MACSPlex data, group comparisons were performed using one-way ANOVA or unpaired t-tests, with multiple comparisons corrected using the Holm method. A p-value < 0.05 was considered statistically significant. Group comparisons and survival analyses were performed using GraphPad Prism (version 10) and MACSPlex data were analyzed in Qlucore Omics Explorer (version 3.10.9; Qlucore AB, Sweden).

## Results

### Patient cohort

**Fig. 1:**
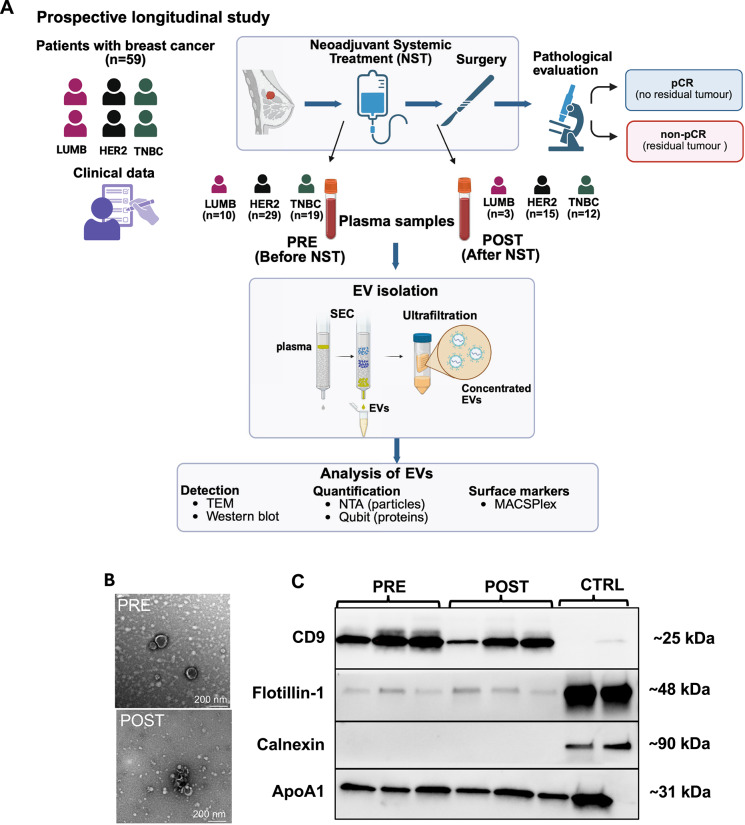
Study design and characterization of plasma-derived extracellular vesicles. **A** Schematic illustration of the study design and workflow. Fifty-nine patients with breast cancer were included in the study. Blood samples were collected at two time points: before NST initiation (PRE) and after completion of NST, prior to surgery (POST). Pathological response was evaluated post-surgery, and patients were classified as either achieving pathological complete response (pCR) or having residual disease (non-pCR). Patients were stratified into breast cancer subtypes (Luminal B-like (LUMB), HER2-positive, and triple-negative breast cancer (TNBC)), and clinical data were recorded. EVs were isolated from 1 mL of plasma using size exclusion chromatography (SEC) followed by ultrafiltration. Downstream EV characterization and analysis included transmission electron microscopy (TEM), western blotting, nanoparticle tracking analysis (NTA), Qubit protein quantification, and MACSPlex surface marker profiling. **B** TEM image showing vesicle-like structures with typical morphology and diameters below 200 nm. Smaller, bright particles were also observed, likely corresponding to co-isolated lipoproteins or other non-vesicular components. **C** Western blot analysis of EV preparations from three patients (PRE and POST). The EV markers CD9 and Flotillin were detected in the EV samples, while the cellular marker Calnexin was absent. The plasma protein ApoA1 was present in all EV preparations, indicating co-isolation of plasma components. Cell lysate was used as a control for CD9, Flotillin and Calnexin, while plasma was used for ApoA. Stain-free SDS PAGE gels used to verify equal loading for Western blot analysis are shown in Additional file 1

A graphical overview of the study protocol is presented in Fig. 1A. Overall, 59 patients with a median age of 51 years were scheduled for NST from January 2019 to September 2020 and were included in the analysis. However, three patients were found to have metastatic disease at baseline radiology (de novo metastatic breast cancer (MBC)). One patient underwent surgery before the completion of NST due to clinical tumor progression. All patients had a minimum follow-up of four years, and the median follow-up was 58 months. The number of patients within each breast cancer subtype were as follows: HER2 + (n = 29), TNBC (*n* = 20) and ER + /HER2- (luminal B, *n* = 10). The median tumor size was 26 mm (range 7–80) and the majority of patients (*n* = 32; 54.2%) had a node-negative disease before NST. The pathological response after NST was evaluable in 56 patients who underwent surgery. Among them, 25 patients (44.6%) achieved a pCR, and 31 patients (55.4%) had residual disease (non-pCR) (Table 1). During follow-up, 13 patients experienced a distant recurrence, and there were no cases of local–regional recurrences. In total, 16 patients were diagnosed with metastatic breast cancer (MBC), including both recurrent and de novo MBC. Eleven patients died from breast cancer.

**Table 1:** Clinicopathological characteristics of the 59 included patients

Variable	
Age (years), median (range)	51 (28–76)
Breast cancer subtypes, *n* (%)	
LUMB	10 (16.9)
HER2	29 (49.2)
TNBC	20 (33.9)
Tumor size (mm), median (range)	26 (7–80)
Tumor size group, *n* (%)	
cT1	19 (32.2)
cT2	29 (49.2)
cT3	9 (15.2)
cTx	2 (3.4)
Axillary metastasis, *n* (%)	
cN0	32 (54.2)
cN1	27 (45.8)
Metastatic disease	
cM0	56
cM1	3
Histological subtype, *n* (%)	
Ductal	56 (94.9)
Lobular	2 (3.4)
Mixed ductal—lobular	1 (8.5)
Ki67 (%), median (range)	55 (11–95)
Grade, n (%)	
I	0
II	35 (60.3)
III	23 (39.7)
Missing	1
Response to NST (*n* = 56^*^), *n* (%)	
Pathological complete response (pCR)	25 (44.6)
Non-pathological complete response (non-pCR)	31 (55.4)

^*^Miller-Payne [30] evaluated in 56 patients, one patient had a clinical progression during NST and underwent surgery before completion of NST, two patients with de novo metastastic BC were not subjected to surgery. Pathological complete response (pCR, Miller-Payne grade 5) and non-pCR (Miller-Payne grade 1–4). cT: size of primary tumour according to mammography and ultrasound, cN: nodal status by ultrasound, cM: clinical or radiological signs of distant metastases, cTx: Tumor size not assessable by clinical or radiological evaluation.

### Characterization of plasma EVs

EVs were successfully isolated from all plasma samples (Fig. 1). EV characterization was performed in accordance with the MISEV 2023 guidelines [11]. The TEM analysis confirmed the presence of vesicle-like structures with typical EV morphology and diameters less than 200 nm (Fig. 1B). Smaller, bright particles were also observed, likely representing co-isolated lipoproteins or other non-vesicular components. Western blot analysis of three representative PRE and POST samples, revealed the expression of EV markers CD9 and Flotillin, and confirmed the absence of Calnexin. However, the lipoprotein ApoA1 was detected in all the EV samples, indicating contamination from plasma proteins. (Fig. 1C). Consistently, the classical EV tetraspanins CD9, CD63, and CD81 were among the most abundantly detected markers in the subsequent MACSPlex analysis (Fig. 3), supporting the presence of typical EV populations. NTA revealed a mean EV concentration of 2.0 × 10^11^ particles/mL in the PRE samples and 2.7 × 10^11^ particles/mL in the POST samples, with a consistent particle-to-protein ratio of 1.1 × 10⁹ particles/µg protein across both time points. Together, these findings confirm that plasma-derived EVs were reliably isolated at both time points, supporting their use in downstream quantitative and surface marker profiling analyses.

### Pre-treatment EV profiles and surface markers

**Fig. 2:**
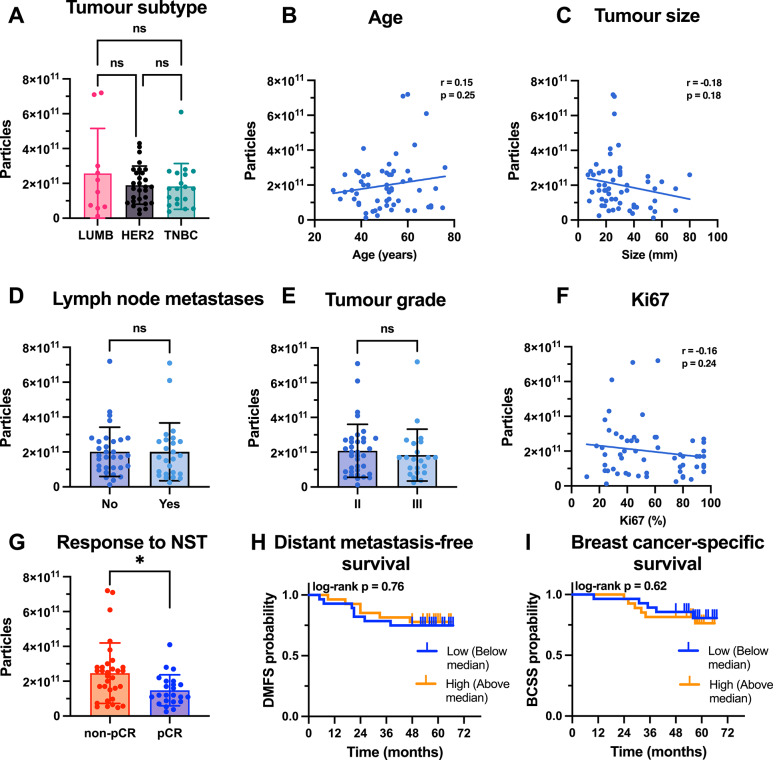
Quantification of plasma-derived EVs isolated from patients with breast cancer before neoadjuvant systemic treatment and correlation with clinical parameters and outcomes. EV particle concentration measured by NTA in PRE plasma samples across **A** breast cancer subtypes: LUMB, HER2 + , and TNBC. (B-F) Correlations between EV particle concentration and clinical variables, including age (**B**), tumor size (**C**), lymph node status (**D**), tumor grade (**E**), and Ki-67 proliferation index (**F**). **G** Comparison of EV concentration between patients who later achieved pathological complete response (pCR) and those who did not (non-pCR). **H**–**I** Kaplan–Meier survival analysis of distant metastasis-free survival (DMFS, H) and breast cancer-specific survival (BCSS, I) based on EV particle concentration, dichotomized at the median (high vs. low). Statistical comparisons were performed using one-way ANOVA (A), unpaired t-tests (G), Pearson correlation (B-F), and log-rank tests (H-I). A *p*-value < 0.05 was considered statistically significant and is indicated by *. Non-significant results are labeled as “ns”. Bar charts show individual values with mean and standard deviation (SD)

Plasma-derived EVs collected at baseline (PRE) were analyzed to assess differences in particle concentration and protein content across breast cancer subtypes, treatment response groups (pCR vs. non-pCR), and clinical parameters (Fig. 2, Additional file 2). EV concentrations in PRE samples varied considerably between patients but did not differ significantly between subtypes (Fig. 2A). There were also no significant correlations between baseline EVs and clinical variables such as age, tumor size, lymph node status, tumor grade, or the Ki-67 index (Fig. 2B-F). However, patients who achieved a pCR had significantly lower EV concentrations at baseline than did those with residual disease (1.5 × 10^11^ vs 2.5 × 10^11^, *p* = 0.016) (Fig. 2G). No significant associations were observed between the PRE EV concentration and distant metastasis-free survival (DMFS) or breast cancer-specific survival (BCSS) (Fig. 2H-I). EV protein content, measured using the Qubit assay, did not differ significantly between patient and tumor characteristics, treatment response groups or DMFS or BCSS. However, a weak negative correlation was observed between baseline protein levels and tumor size (r =  − 0.30, *p* = 0.03) (Additional file 2A-I). To further explore potential subtype-specific effects, we analyzed baseline EVs within LUMB, HER2-positive and TNBC subgroups. These analyses did not reveal additional associations beyond the overall results, apart from confirming the negative correlation between EV protein levels and tumor size in HER2-positive patients. The trend of lower baseline EV concentration in pCR compared with non-pCR was also preserved in HER2-positive and TNBC patients, whereas all LUMB patients were non-pCR (Additional file 3A-B).

**Fig. 3:**
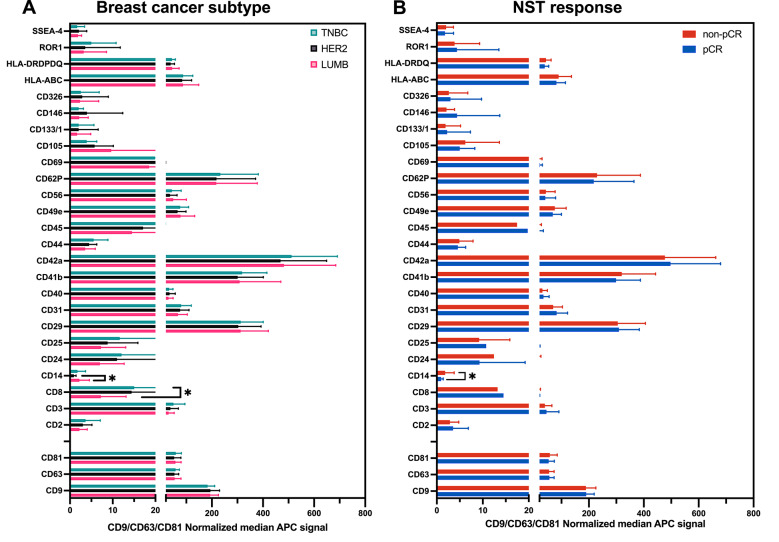
Pre-treatment EV surface marker profiles across breast cancer subtypes and treatment response groups. Surface marker expression in plasma-derived EVs collected before neoadjuvant systemic treatment (PRE), analyzed using the MACSPlex assay. Values represent CD9/CD63/CD81-normalized median fluorescence intensity (MFI, APC channel) for markers retained after threshold filtering (MFI > 0.5 in ≥ 50% of samples). **A** Marker expression grouped by breast cancer subtype: Luminal B (LUMB), HER2-positive (HER2 +), and triple-negative breast cancer (TNBC). **B** Marker expression grouped by treatment response: pathological complete response (pCR) vs. non-pCR. Note that all EV samples were collected before treatment, while response was evaluated after completion of NST. Bars represent mean values with standard deviation (SD). Statistical comparisons were performed using one-way ANOVA or unpaired t-tests, with multiple comparisons corrected using the Holm method. A *p*-value < 0.05 was considered statistically significant and is indicated by *; non-significant results are labeled as “ns”. Individual marker data are shown in Additional files 5 (subtypes) and 6 (treatment response)

The EV surface marker expression was assessed using the MACSPlex assay. After normalization to CD9, CD63, and CD81, as well as threshold filtering (MFI > 0.5 in ≥ 50% of the samples), 28 out of 37 markers were retained for analysis (Fig. 3). Markers were grouped based on known associations with cellular origin and biological function, including platelet-associated, immune-associated, endothelial/stromal-associated, tumor/cancer-associated, classical EV markers (tetraspanins), stem/progenitor-associated, and the broadly expressed cancer/stromal/immune-associated markers (Additional file 4). Platelet-associated markers (CD41b, CD42a, and CD62P) accounted for the largest proportion of the total EV surface signal, followed by broadly expressed markers (CD29, CD105, and CD44), tetraspanins (CD9, CD63, and CD81), endothelial/stromal markers (CD49e and CD31), and immune-associated markers (e.g., HLA-ABC, CD3, and CD14). In contrast, markers associated with stem/progenitor-like cells (CD133/1 and SSEA-4) and tumor/cancer-associated proteins (CD24, CD146, CD326, and ROR1) presented lower overall expression across the cohort (Additional file 4).

Only two markers differed significantly between breast cancer subtypes: CD8 was more highly expressed in TNBC samples than in luminal B-like samples, whereas CD14 was more abundant in luminal B-like samples than in HER2-positive samples (Fig. 3A, Additional file 5). No consistent subtype-specific clustering was observed across the remaining panel. When stratified by eventual pathological response to NST, CD14, although detected at low levels, was significantly highly expressed at baseline in patients with non-pCR (Fig. 3B, Additional file 6). This difference appeared to be largely driven by luminal B-like patients, all of whom were non-pCR, whereas no significant differences were observed within the HER2-positive or TNBC subgroups (Additional file 7 A-C). Although not statistically significant after correction for multiple testing, CD24 showed the largest numerical difference, with higher expression in patients with non-pCR, although the variability between individual patients was high. This trend was also noted within the HER2-positive and TNBC subgroups (Additional file 7A-C). Taken together, these findings suggest that EV concentration and specific surface marker profiles in samples before NST may be associated with subsequent treatment response and reflect baseline variation in tumor- and immune-derived EV composition.

### Post-treatment EV profiles and associations with clinical outcome

**Fig. 4:**
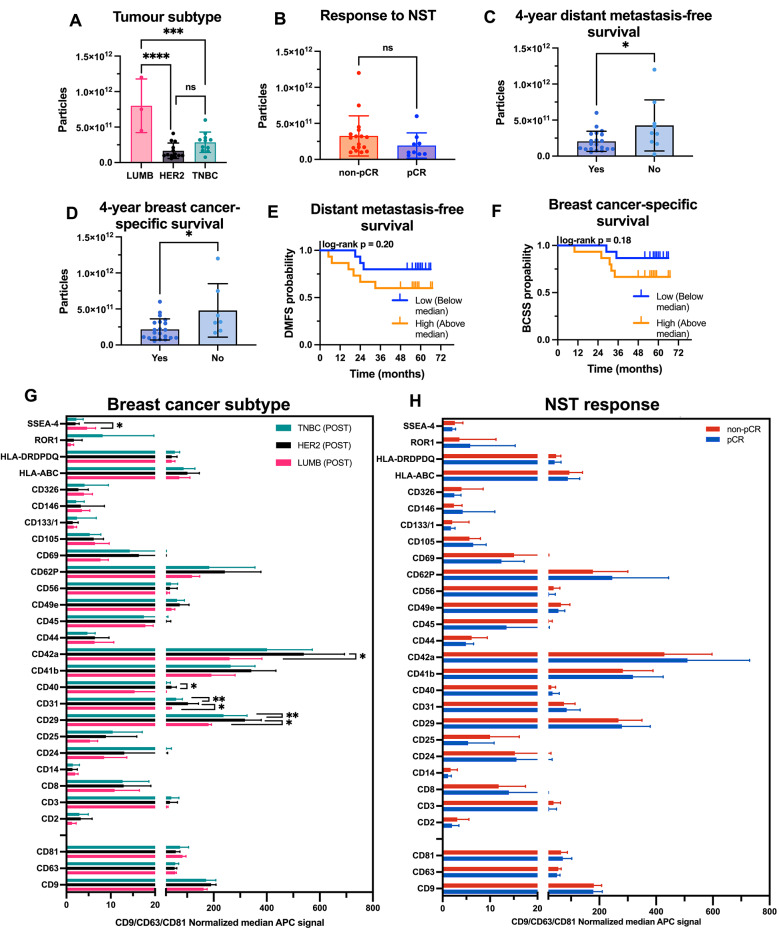
Characterization of plasma-derived EVs isolated after neoadjuvant systemic treatment and correlation with clinical outcomes. EV particle concentration (measured by NTA) in POST plasma samples across breast cancer subtypes: Luminal B (LUMB), HER2-positive (HER2 +), and triple-negative breast cancer (TNBC). (B-D) EV concentration in relation to pathological treatment response (**B**), metastatic recurrence (**C**), and breast cancer-specific mortality within four years (**D**); “Yes” = event-free, “No” = event occurred. **E**–**F** Kaplan–Meier survival analysis of distant, based on POST EV concentration dichotomized at the median (high vs. low). **G** EV surface marker expression in POST samples grouped by breast cancer subtype. (H) EV surface marker expression in POST samples grouped by treatment response (pCR vs. non-pCR). All surface marker data are shown as CD9/CD63/CD81-normalized MFI (APC channel), for markers retained after threshold filtering (MFI > 0.5 in ≥ 50% of samples). Statistical comparisons were performed using one-way ANOVA (A, G), unpaired *t*-tests (B-D, H), and log-rank tests (E–F). A *p*-value < 0.05 was considered statistically significant and is indicated as follows: *p < 0.05; **p < 0.01; ***p < 0.001; ****p < 0.0001; “ns” = not significant. Bar charts show individual values with mean and standard deviation (SD)

EVs isolated after neoadjuvant systemic treatment (POST) were analyzed according to breast cancer subtype, treatment response, and clinical outcomes (Fig. 4, Additional files 8–9). The EV particle concentration, measured by NTA, was significantly greater in patients with Luminal B-like subtype compared to patients with TNBC or HER2-positive disease (Fig. 4A). A similar trend to that observed in PRE samples was noted when stratified by treatment response: patients with non-pCR had higher EV concentrations than those who achieved pCR did, although this difference did not reach statistical significance (Fig. 4B). Thus, while post-treatment EV concentrations did not significantly distinguish pCR from non-pCR across the full cohort, Luminal B-like tumors showed the highest post-treatment EV levels overall, consistent with their lower pCR rates and higher residual disease burden. At the four-year landmark analysis, post-treatment EV concentrations were higher in patients who later developed a distant metastatic recurrence (Fig. 4C) and in those who died of breast cancer, compared with patients who remained event-free (Fig. 4D). However, these differences did not reach statistical significance in time-event analysis (Fig. 4E–F). No significant associations were observed between EV concentration and tumor size, lymph node status, or tumor grade (data not shown).

EV surface marker expression in POST samples, as assessed via the MACSPlex assay, was significantly different between breast cancer subtypes. In patients with HER2-positive breast cancer, CD40 was more highly expressed than in patients with TNBC, CD42a was more highly expressed than in patients with Luminal B-like breast cancer, and both CD31 and CD29 were more highly expressed than in patients with both Luminal B-like breast cancer and TNBC (Fig. 4G, Additional file 8). In contrast, SSEA-4 was expressed at higher levels in patients with luminal B-like disease than in HER2-positive patients. These markers represent diverse biological functions, including platelet activation (CD42a), immune regulation (CD40), endothelial or stromal signaling (CD31), and cell adhesion (CD29). When stratified by treatment response, no markers were significantly different between the pCR and non-pCR groups (Fig. 4H, Additional file 9). Although not statistically significant, several markers, including CD326, CD14, CD25, and CD45, tended to be more highly expressed in patients with non-PCR. These markers are associated with tumor- or immune-related EVs. This trend may suggest underlying differences in EV content between response groups but should be interpreted with caution.

### Changes in EV profiles during neoadjuvant systemic treatment

**Fig. 5:**
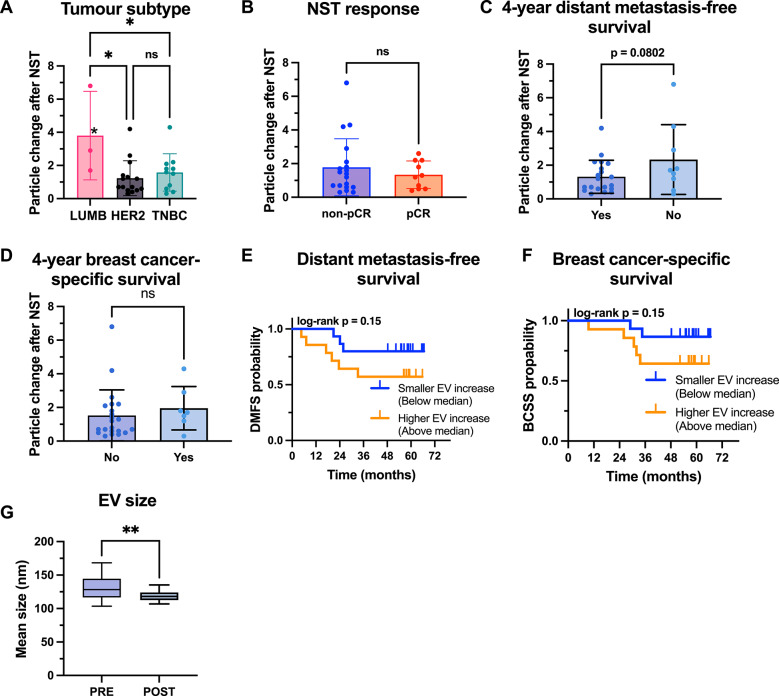
Changes in EV particle concentration and size following neoadjuvant systemic treatment. (A-F) POST-to-PRE fold change in EV particle concentration (measured by NTA), analyzed in relation to clinical subgroups and outcomes. **A** Fold change grouped by breast cancer subtype: Luminal B (LUMB), HER2-positive (HER2 +), and TNBC. **B** Fold change grouped by treatment response: pathological complete response (pCR) vs. non-pCR. (C-D) Fold change grouped by **C** 4-year distant metastasis status and **D** breast cancer-related death, where “Yes” = event-free, “No” = event occurred. **E**, **F** Kaplan–Meier survival analysis of distant metastasis-free survival (DMFS, E) and breast cancer-specific survival (BCSS, F), based on fold change dichotomized at the median. **G** Paired comparison of EV particle size (mode) before and after NST. Statistical comparisons were performed using one-way ANOVA (A), unpaired t-tests (B-D), log-rank tests (E–F), and paired t-tests (G). A *p*-value < 0.05 was considered statistically significant and is indicated by *. In panel A, asterisks above bars indicate significant within-group differences (POST vs PRE), while asterisks between bars denote significant differences between subtypes. Non-significant comparisons are labeled “ns”. Bar charts show mean values with individual patient dots and standard deviation (SD)

**Fig. 6:**
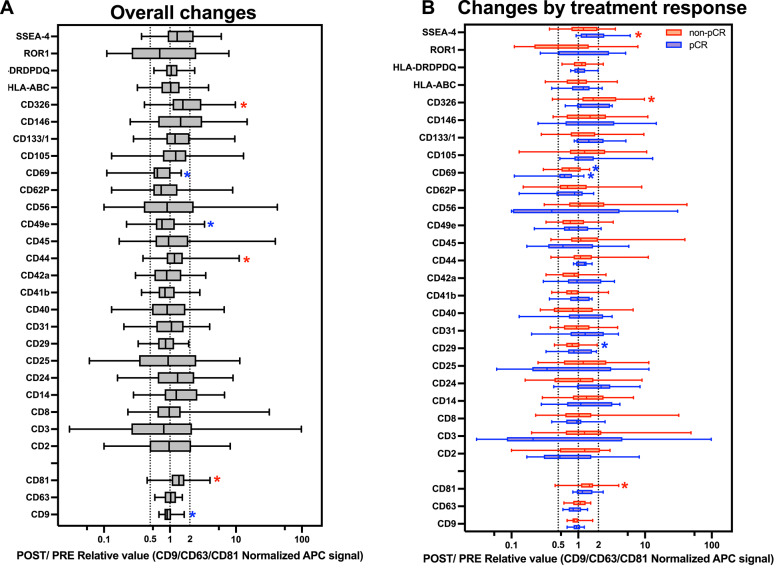
EV surface marker expression changes in response to neoadjuvant systemic treatment. Surface marker expression in plasma-derived EVs before and after neoadjuvant systemic treatment (NST), assessed using MACSPlex analysis. For each patient and marker, data are presented as the POST/PRE ratio of CD9/CD63/CD81-normalized APC signal, representing the individual fold change. **A** All patients combined. **B** Stratified by treatment response: pathological complete response (pCR) vs. non-pCR. Graphs show show box plots with median, interquartile range, and full range. All data are plotted on a log scale; values > 1 indicate increased expression post-treatment, and values < 1 indicate decreased expression. Statistical comparisons were performed using paired t-tests with Holm correction. Colored asterisks indicate significant within-group changes relative to baseline (PRE), red asterisks indicate significant upregulation post-treatment, and blue asterisks indicate significant downregulation post-treatment. *Significance level: *p < 0.05

To evaluate treatment-induced changes in EV profiles, PRE and POST samples were compared within patients. EV particle concentration, particle size, and surface marker expression were assessed, and changes were further examined in relation to breast cancer subtype, treatment response, and clinical outcomes (Figs. 5 and 6, Additional files 10–12). The EV particle concentration increased in the majority of patients after treatment (Fig. 5A), although the degree of change varied substantially. A statistically significant increase in EV particle concentration was observed within the luminal B-like group, whereas no change was detected in patients with HER2-positive or TNBC breast cancer (Additional file 10A). In addition, the fold change was significantly greater in patients with luminal B-like breast cancer than in both the HER2-positive and TNBC groups (Fig. 5A). The POST/PRE fold change in EV particle concentration did not differ significantly between patients who achieved a pCR and those who did not (Fig. 5B).

Patients who developed distant metastatic recurrence tended to have a greater POST/PRE fold change in EV concentration than patients without recurrence did, although this difference was not statistically significant (*p* = 0.08, Fig. 5C). No differences were found when patients with and without breast cancer-related death were compared (Fig. 5D). There were no statistically significant associations between the EV fold change and distant metastasis-free survival or breast cancer-specific survival (Fig. 5E-F), although patients with above-median fold changes tended to have poorer outcomes (*p* = 0.15). Finally, a small but statistically significant decrease in the mean EV particle size was observed following treatment (Fig. 5G).

We then performed EV surface marker analysis. Among the 28 markers retained after threshold filtering, several showed statistically significant changes following NST. CD326, CD44, and CD81 were significantly upregulated, whereas CD69, CD49e, and CD9 were significantly downregulated in POST samples compared with PRE samples (Fig. 6A, Additional file 11). These results suggest that NST induces measurable changes in EV surface marker expression across the cohort. Marker expression was then examined within each breast cancer subtype. Paired comparisons between the POST and PRE samples revealed multiple significant within-group changes, particularly in patients with HER2-positive breast cancer (Fig. 6B, Additional file 12). Five markers, namely, CD29, CD31, CD40, CD41b, and CD42a, were significantly greater in patients with HER2-positive disease than in those with luminal B-like and/or TNBC (Additional file 12). A similar analysis was performed on the basis of treatment response. Within both the pCR and non-pCR groups, several markers showed significant changes from PRE to POST (Fig. 6B, Additional file 11). When comparing the fold changes between response groups, some markers, including CD326, tended to show greater increases in patients with non-pCR. However, these differences were not statistically significant (Fig. 6B).

## Discussion

In this study, we present the characterization of EVs from the prospectively analyzed paired plasma samples from patients with breast cancer undergoing NST to explore the clinical relevance of EVs as potential biomarkers of therapy response. Our results indicate that EVs reflect underlying tumor biology and EV levels relate to clinical outcome and treatment response, such that patients who achieved pCR had lower EV levels in the baseline plasma samples compared to those who experienced residual disease. Consistent with our results, patients who remained recurrence-free and those with superior breast cancer-specific survival tended to have lower post-treatment EV levels. While these differences were apparent at the four-year landmark, they did not reach statistical significance in Kaplan–Meier analyses, likely reflecting the limited power of our cohort. These findings should therefore be regarded as hypothesis-generating. In addition to concentration changes, several EV surface markers, most notably EpCAM and SSEA-4, were differentially expressed depending on treatment response, whereas other markers changed more uniformly across the cohort. Together, these results suggest that EV profiles may reflect both therapy-induced effects and residual disease biology, supporting their potential relevance as dynamic, non-invasive biomarkers. In line with this interpretation, elevated EV concentrations may reflect more aggressive tumor behavior or a greater burden of active tumor-host interactions. This interpretation is supported by previous reports, including a study by König et al., which demonstrated that elevated EV concentrations, both before and after NST, were associated with poorer response and survival in patients with breast cancer [25]. However, as our cohort was relatively small, these findings should be considered exploratory.

The observed association between lower EV levels and favorable outcomes supports the potential relevance of EV quantification as a non-invasive, dynamic approach to monitor treatment efficacy and stratify risk. Importantly, the weak and negative correlation between EV concentration and tumor size indicates that EV burden is not merely a proxy for tumor volume but may instead reflect biologically relevant features of tumor activity and microenvironmental interactions. Taken together, these findings should be interpreted cautiously and considered hypothesis-generating until validated in larger cohorts.

Surface marker profiling using the MACSPlex technique revealed a broad expression pattern, with platelet- and broadly expressed stromal/cancer/immune and EV markers being most abundant. Interestingly, CD14 was detected at low levels and appeared more prevalent in patients with non-pCR, but this difference was not observed within HER2-positive or TNBC patients and appeared to be mainly driven by the luminal B subgroup, in which all patients were non-pCR. This finding is consistent with the higher residual disease burden typically seen in luminal B like tumours and may reflect subtype-specific treatment response rather than intrinsic aggressiveness. In contrast, CD8 expression was higher in patients with TNBC. These findings suggest that EVs mirror immune cell infiltration and activation status, which are increasingly recognized as important determinants of the response to NST, particularly in immunogenic subtypes such as TNBC [31]. The lack of clear baseline differences between subtypes is likely explained by the limited panel of 37 markers available in the MACSPlex assay, together with the relatively small number of patients in each subgroup.

EV surface profiling revealed both general and response-specific effects of treatment. Across the cohort, expression of several markers changed consistently following NST, regardless of response. CD69, CD29 (integrin β1), and CD49e (integrin α5) were all significantly reduced, particularly in non-pCR patients. CD69 is an early activation marker of lymphocytes and is involved in tissue retention, Treg/Th17 differentiation, and immune modulation [32]. CD29 (integrin β1) and CD49e (integrin α5) form the α5β1 integrin heterodimer, a key fibronectin receptor that mediates adhesion to the extracellular matrix (ECM). This integrin complex is widely expressed on stromal and immune cells and is frequently overexpressed in breast cancer, where it has been associated with invasion, metastasis, and poor prognosis [33]. Moreover, α5β1 has been shown to be enriched on EVs derived from metastatic breast cancer cells, where it may promote tissue-specific uptake by stromal cells and contribute to pre-metastatic niche formation [33, 34]. These integrins were among the most abundant EV markers in our dataset, and their coordinated downregulation may reflect reduced EV shedding from breast cancer cells or ECM-interacting stromal cells following NST.

CD44 was significantly upregulated after NST and was particularly elevated in some non-pCR patients. As an adhesion receptor involved in ECM interactions and cellular plasticity, CD44 is frequently associated with cancer progression, stemness, and residual tumor adaptation [35–37]. Its increase may reflect remodeling of the tumor microenvironment or persistent activity of aggressive tumor cell populations, as well as continued release of tumor-derived EVs after treatment.

Although EVs did not significantly distinguish pCR from non-pCR across the full cohort, subtype-specific trends were evident. Luminal B-like tumors showed both the highest post-treatment EV concentrations and the largest relative increase during therapy, consistent with their lower chemosensitivity compared with HER2-positive and TNBC subtypes. These findings suggest that EV dynamics are consistent with aspects of treatment-resistant tumor biology rather than intrinsic subtype aggressiveness. The results should therefore be regarded as hypothesis-generating and require validation in larger cohorts.

When the data were stratified by treatment response, two markers showed divergent patterns. Post-treatment, CD326 (EpCAM) was selectively upregulated in non-pCR patients but not in pCR patients. EpCAM is an epithelial adhesion molecule that is overexpressed in various cancers and is associated with tumor aggressiveness and therapeutic resistance [38]. While baseline levels were similar between groups, the post-treatment elevation in non-responders suggests continued shedding of EpCAM + EVs from residual tumor tissue, highlighting its potential as a dynamic marker of incomplete response. Conversely, SSEA-4 (stage-specific embryonic antigen-4), a glycosphingolipid expressed on embryonic stem cells, mesenchymal progenitors, and cancer stem-like cells [39, 40], was significantly upregulated in pCR patients only. This may reflect treatment-induced tissue remodeling or activation of regenerative progenitor-like cells in patients receiving therapy.

Not all regulated markers were equally abundant. Stromal and adhesion-related markers such as CD29 and CD49e accounted for a substantial proportion of the total EV signal, whereas tumor-associated markers such as EpCAM and SSEA-4 were expressed at lower levels. Despite this, their selective modulation in relation to treatment outcome suggests biological and potential clinical relevance.

Longitudinal analysis of matched samples revealed a statistically significant decrease in EV particle size following treatment, whereas the EV particle concentration generally increased following NST, particularly in patients with luminal B-like tumors. This group is generally less sensitive to chemotherapy and the higher post-treatment EV concentrations observed in luminal B patients may therefore reflect their lower response rate and higher burden of residual disease compared with HER2 + and TNBC, rather than baseline tumor aggressiveness. While an overall increase in EV concentration may reflect therapy-induced cellular stress or immune activation, the subtype-specific pattern suggests intrinsic differences in how tumors modulate EV release during treatment. Greater changes in EV concentrations post-treatment tended to be associated with worse distant metastasis-free survival and breast cancer-specific survival, although these associations did not reach statistical significance, likely due to limited power. Nevertheless, this trend supports the premise that dynamic monitoring of EVs, rather than single-time point assessments, may provide greater insight into treatment response and prognosis.

In addition to biological factors, treatment-related effects may also influence EV profiles. The EV profiles are likely to be modulated by the specific neoadjuvant treatment regimens administered across different breast cancer subtypes [41–43]. Our study was not powered or designed to model treatment related effects on EV abundance or lack thereof. We acknowledge the potential confounding introduced by the differences in the neoadjuvant therapies on post-treatment EV profiles. Prospective studies powered to detect treatment related differences will help understand therapy-independent effects on EV biology.

A key strength of our study is the use of a prospectively collected, clinically representative cohort of aggressive breast cancer subtypes, with standardized protocols for EV isolation, analysis, achieving high procedural consistency. Characterization was performed according to the MISEV2023 guidelines [11] and included NTA, TEM, Western blot, protein quantification, and surface marker profiling, ensuring robust EV identification.

This study has several limitations. First, the relatively small sample size, particularly within subgroups, limits statistical power and the ability to detect subtle but clinically meaningful differences. Therefore, the findings should be considered hypothesis-generating and require validation in larger, independent cohorts. Second, the MACSPlex platform, while enabling standardized multiplex detection of 37 surface markers, does not allow detection of intravesicular proteins or nucleic acids and may miss relevant markers outside the fixed panel. Given the relatively large cohort size compared with many previous EV studies, the limited plasma volumes and the modest purity of plasma-derived EVs, we considered MACSPlex the most suitable approach, as it enables standardized and multiplex profiling from small input material across a large number of samples. A third limitation concerns the purity of the EV preparations. Although SEC is widely used due to its reproducibility and gentle handling, it may co-isolate non-EV components such as lipoproteins and abundant plasma proteins [44].

While these contaminants are unlikely to affect surface marker detection via MACSPlex, they may contribute to background signals in concentration or protein quantification assays (e.g., NTA and Qubit). SEC was chosen for this study due to its compatibility with small plasma volumes and the need for parallel, standardized processing of multiple patient samples. However, future studies may benefit from incorporating additional purification steps to increase EV purity. Additionally, samples were collected before EV-specific pre-analytical protocols were fully implemented, and plasma was only centrifuged once before freezing, which may have led to the presence of residual platelets or platelet-derived EVs. Importantly, recent guidelines highlight that it is technically challenging to completely distinguish platelet-derived EVs from residual platelets or fragments, since their size, density, and surface marker profiles overlap [45]. However, all samples underwent a second centrifugation step prior to SEC and were processed uniformly, reducing the risk of systemic bias. The strong platelet- and immune-associated signals observed in our MACSPlex profiling are therefore most consistent with EVs derived from hematopoietic cells, which are known to dominate plasma. This predominance of hematopoietic cell-derived EVs likely explains why tumor-associated markers were relatively underrepresented in our profiling. Importantly, this is not unexpected, as tumor-derived EVs constitute only a small fraction of the total circulating EV pool [46]. Rather than reflecting a technical limitation alone, our results capture the broader systemic EV landscape in plasma, integrating both tumor-derived signals and host responses that may carry complementary clinical relevance.

Although healthy control samples were not available, the use of paired pre- and post-treatment samples allowed for within-patient comparisons, thereby improving the ability to evaluate treatment-related EV changes. However, this limits interpretation relative to EV profiles in non-cancer individuals. Finally, a general challenge in the EV field is the inability to reliably determine the cellular origin of EVs in plasma, which contains a complex mixture of vesicles released from multiple cell types. These include not only carcinoma cells, but also cells from the surrounding stromal and immune compartments of the tumor microenvironment, as well as circulating blood and endothelial cells. Although surface markers such as CD326 and CD24 suggest epithelial origin, their expression is not exclusive to tumor cells as multiple epithelial cell types may express these markers. The presence and modulation of such markers in response to neoadjuvant treatment, however, suggest that at least a subset of the plasma-derived EVs may originate from tumor cells. Future studies employing more specific or integrative approaches (e.g., EV-RNA sequencing or imaging flow cytometry) may improve our ability to distinguish the cellular sources of EVs in plasma. In addition, emerging computational deconvolution methods that integrate EV surface profiling with single-cell or bulk omics data may offer further resolution to distinguish tumor-derived EVs from stroma-derived or immune-derived EVs in complex patient samples.

## Conclusion

This study illustrates the feasibility and potential utility of plasma-derived EV profiling to provide non-invasive insights into treatment response and tumor biology in patients with breast cancer. Our findings highlight the potential value of EVs as dynamic biomarkers and support further investigation in larger cohorts. Future studies should integrate EV profiling with other liquid biopsy components, such as ctDNA, and explore functional assays to elucidate the biological impact of EVs on therapy resistance and metastasis.

## Supplementary Information

Below is the link to the electronic supplementary material.

Supplementary Material 1

## Data Availability

Raw data that support the findings of this study are available from the corresponding author upon reasonable request. All relevant data of experiments have been deposited in the EV‐TRACK knowledgebase [47] with the EV‐TRACK‐ID EV250083.

## Abbreviations

APC: Allophycocyanin
BC: Breast cancer
BCSS: Breast cancer-specific survival
CD: Cluster of differentiation
DMFS: Distant metastasis-free survival
ER: Estrogen receptor
EV: Extracellular vesicle
FITC: Fluorescein isothiocyanate
HER2: Human epidermal growth factor receptor 2
IO: Immuno-oncology
MFI: Median fluorescence intensity
MISEV: Minimal information for studies of extracellular vesicles
NTA: Nanoparticle tracking analysis
NST: Neoadjuvant systemic treatment
PE: Phycoerythrin
PBS: Phosphate-buffered saline
pCR: Pathologic complete response
PRE: Pre-treatment (before NST)
POST: Post-treatment (after NST)
SEC: Size exclusion chromatography
TEM: Transmission electron microscopy
TNBC: Triple-negative breast cancer

## Declarations

### Generative AI use

During the preparation of this work, the authors used ChatGPT (OpenAI, GPT-4) to assist with improving language clarity, grammar, and scientific expression. The authors reviewed and edited all AI-generated content and accept full responsibility for the published material.
