# Supplementary material for: Plasma extracellular vesicles reflect response and prognosis in patients with breast cancer undergoing neoadjuvant treatment
Source: Breast Cancer Res. 2026 Jan 9;28:22. doi: 10.1186/s13058-025-02209-0 (PMC12849309; doi:10.1186/s13058-025-02209-0)
Supplement: Supplementary file 1 — Supplementary Material 1 [file 13058_2025_2209_MOESM1_ESM.pdf]

Additional file 1

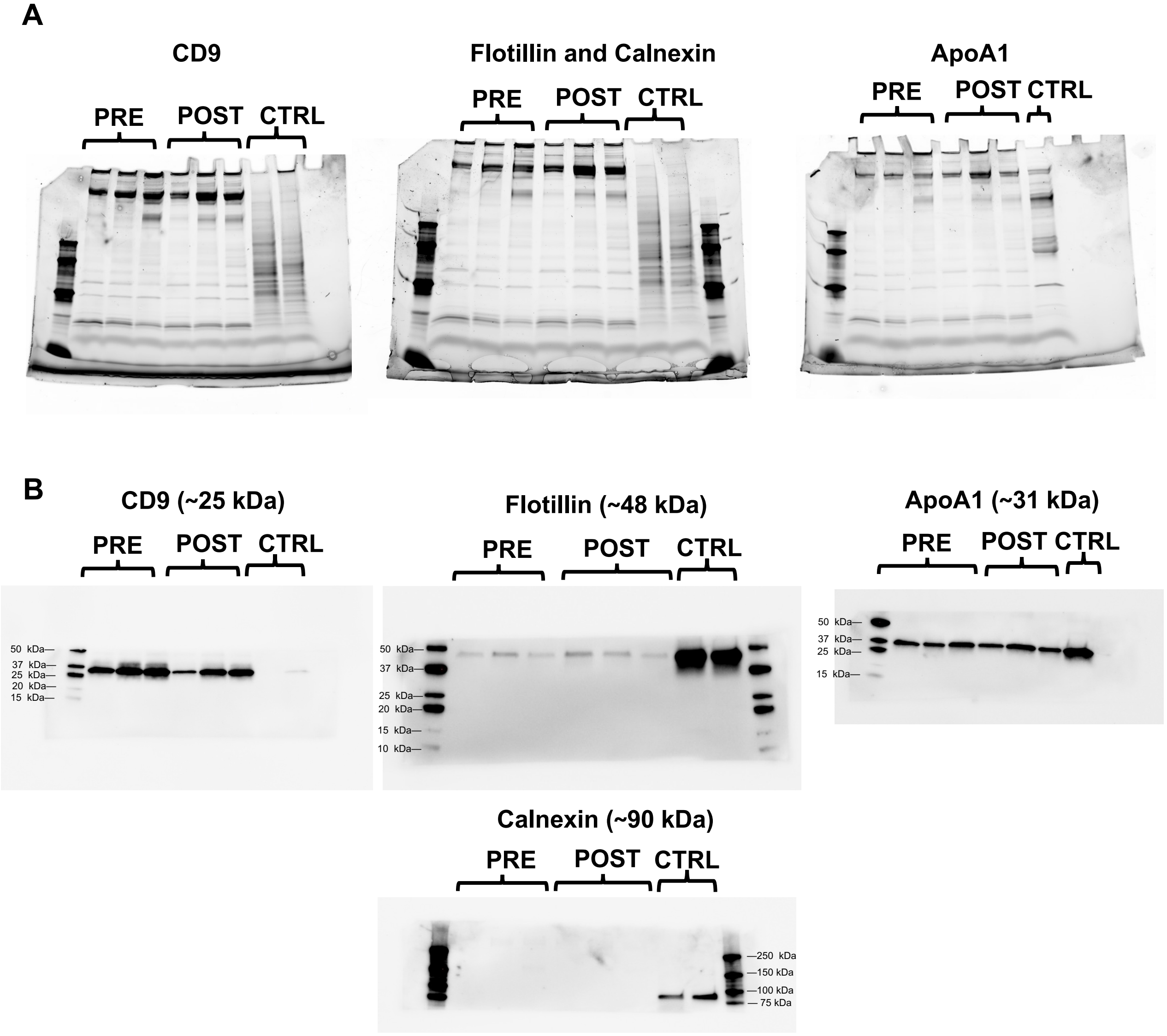

Additional file 1. Uncropped SDS-PAGE gels and Western blots.

(A) Uncropped SDS-PAGE gels (post-run, pre-transfer) and (B) uncropped Western blots for CD9, Flotillin, Calnexin and ApoA1. Gels were imaged immediately after electrophoresis and before protein transfer. Samples include pre-NST and post-NST conditions with positive controls for CD9, Flotillin and Calnexin were cell lysate, while plasma was used for ApoA1. Cropped versions of these blots are shown in Figure 1C.

Additional file 2

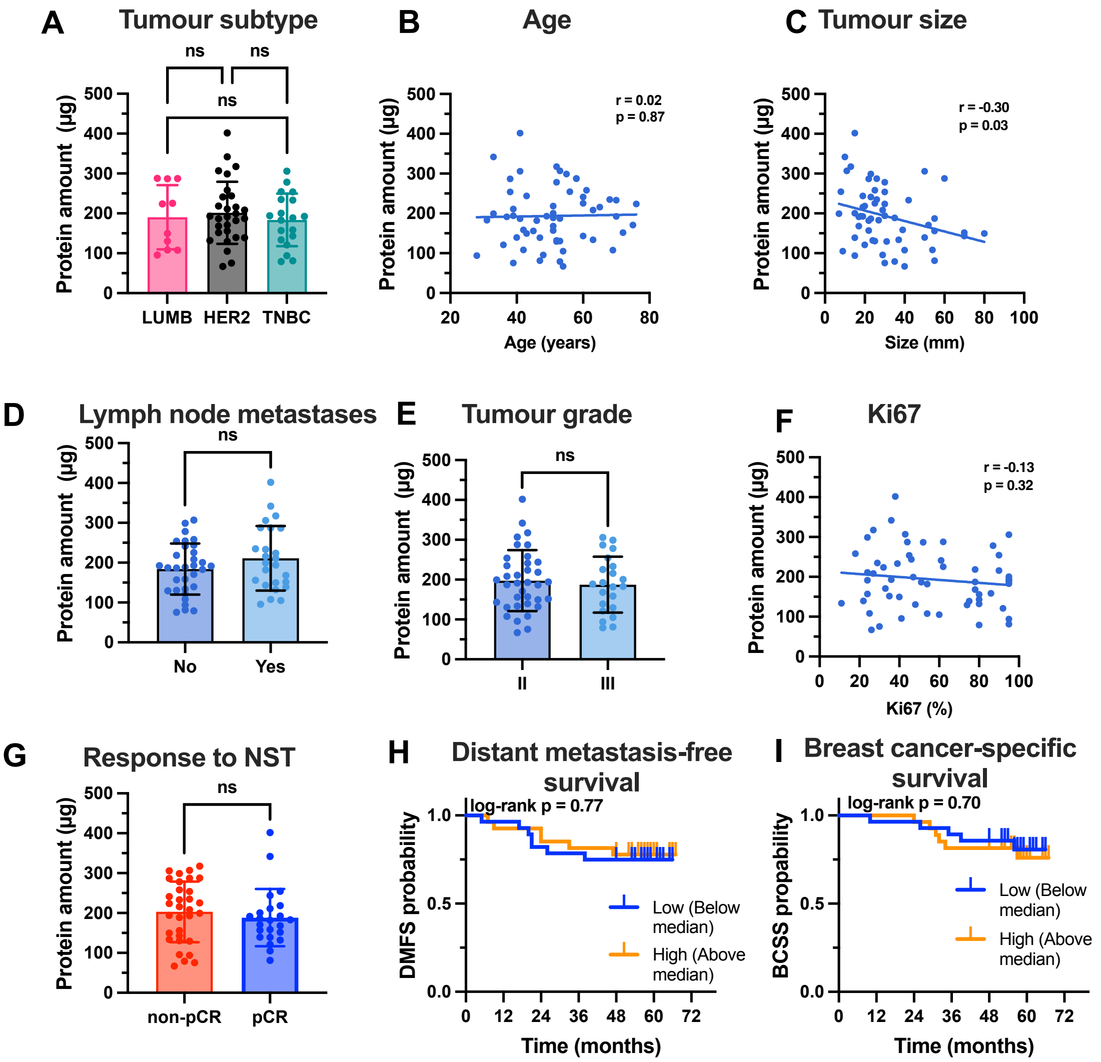

Additional file 2. EV protein amount in PRE samples and correlation with clinical parameters.

EV protein concentration was measured using the Qubit assay in EVs isolated from 1 mL of plasma. The resulting protein amounts per sample are shown across subtypes and clinical parameters. (A) Protein concentration across breast cancer subtypes: luminal B (LUMB), HER2-positive (HER2+), and triple-negative breast cancer (TNBC). (B-F) Correlations with clinical parameters: age (B), tumor size (C), lymph node status (D), tumor grade (E), and Ki-67 index (F). (G) Comparison between patients who later achieved pathological complete response (pCR) and those with residual disease (non-pCR). (H-I) Kaplan-Meier survival analysis of distant metastasis-free survival (DMFS, H) and breast cancer-specific survival (BCSS, I), based on protein concentration dichotomized at the median.

Statistical tests: one-way ANOVA (A), unpaired t-tests (G), Pearson correlation (B-F), and log-rank tests (H-I). A p-value < 0.05 was considered statistically significant and is indicated by *; non-significant comparisons are labeled “ns”. Bar charts show mean ± standard deviation (SD) with individual data points.

Additional file 3

A

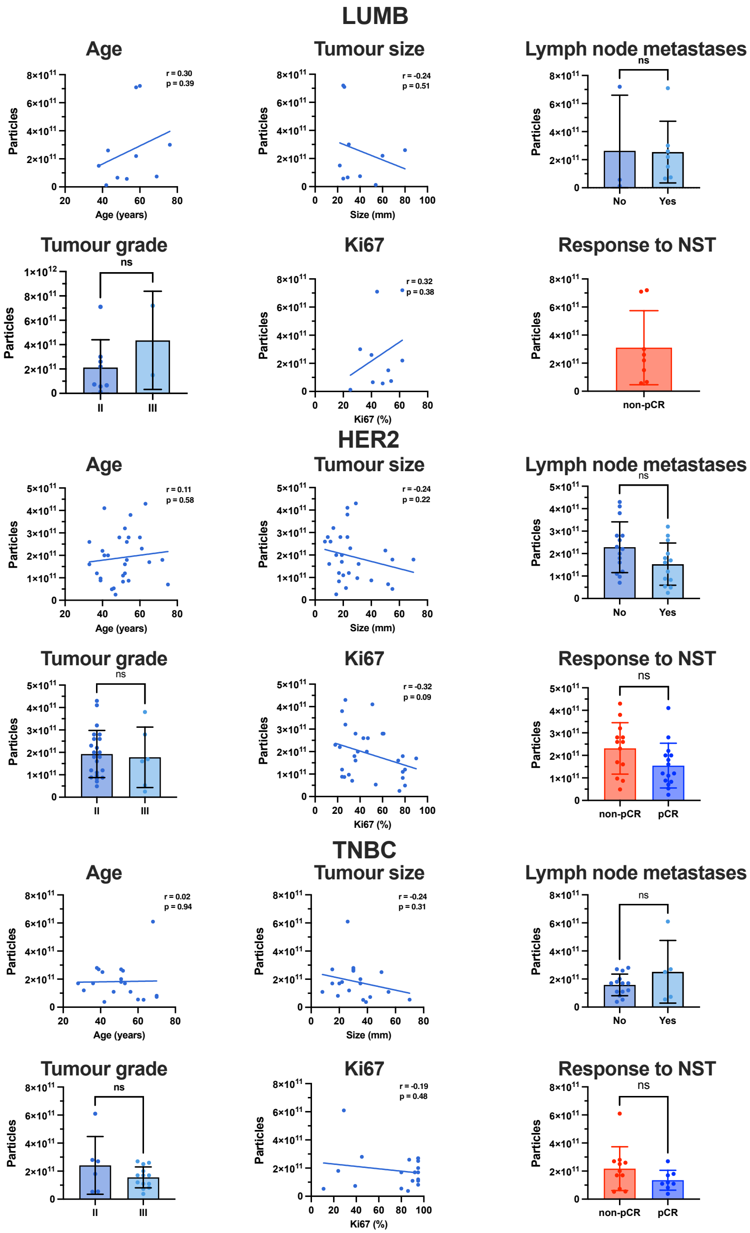

Additional file 3. Quantification of PRE samples within breast cancer subtypes and correlation with clinical parameters.

(A) Baseline EV particle concentration (NTA) and (B) protein content (Qubit) analyzed within breast cancer subtypes: luminal B-like (LUMB), HER2-positive (HER2+), and triple-negative breast cancer (TNBC). Panels correspond to the same analyses as Figure 2B-G (particle concentration) and Additional file 2A–G (protein), but are shown separately by subtype: correlations with clinical parameters (age, tumor size, lymph node status, tumor grade, and Ki-67) and comparison by pathological response (pCR vs. non-pCR).
Statistical tests: unpaired t-tests (lymph node status, tumor grade, and response to NST) and Pearson correlation (age, tumor size, and Ki-67). For correlation analyses, both the correlation coefficient (r) and p-value (p) are displayed in the graphs. A p-value <0.05 was considered statistically significant and is indicated by *; non-significant comparisons are labeled “ns”. Bar charts show mean ± standard deviation (SD) with individual data points.

Additional file 4

A

Cancer/stromal/immune-associated	CD29, CD105, CD44
Endothelial-/stromal-associated	CD49e, CD31
Classical EV markers (tetraspanins)	CD9, CD63, CD81
Immune-associated	HLA-ABC, HLA-DRDPDQ, CD2, CD3, CD56, CD40, CD69, CD45, CD8, CD25, CD14
Platelet-associated	CD42a, CD41b, CD62P
Stem-/progenitor-associated	SSEA-4, CD133/1
Tumor-specific or cancer-associated	ROR1, CD24, CD326, CD146

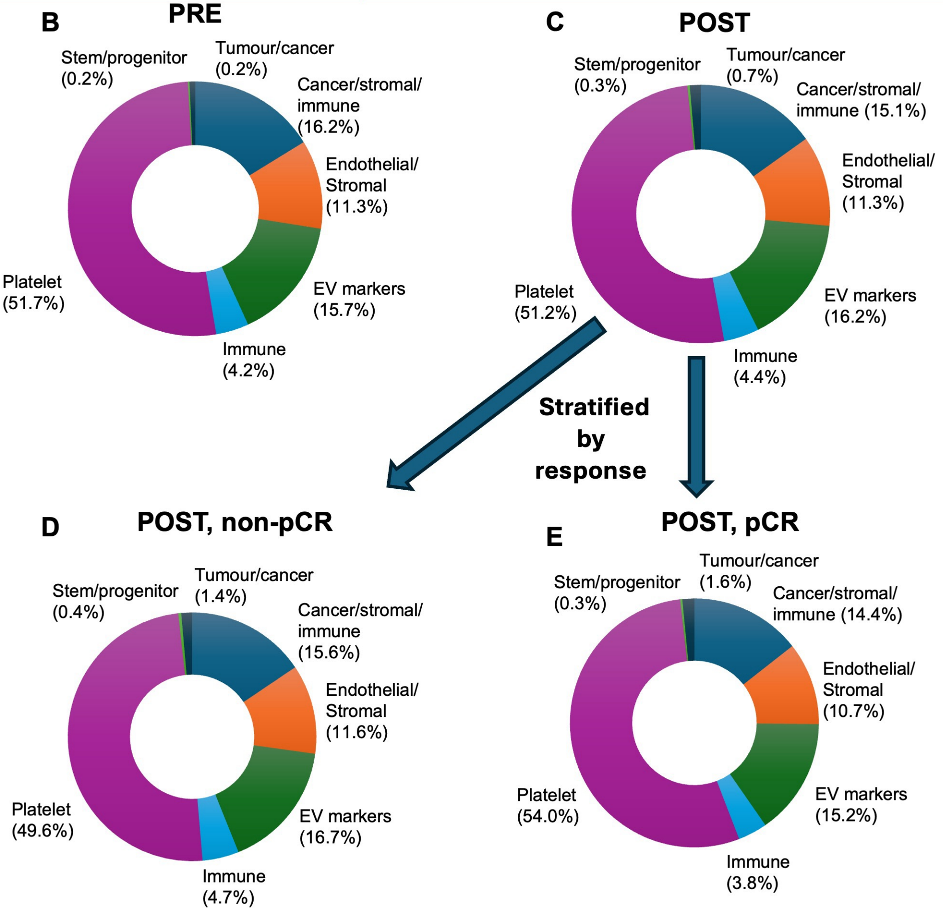

Additional file 4. Functional grouping and relative signal distribution of EV surface markers.

A) Clustering of the 28 MACSPlex markers into seven categories based on known cellular origin and biological function: platelet-associated, immune-associated, endothelial/stromal-associated, tumor/cancer-associated, classical EV markers (tetraspanins), stem/progenitor-associated, and broadly expressed cancer/stromal/immune-associated markers. (B-E) Donut plots displaying the contribution of each group to the total EV surface marker signal. Group-level signal was calculated as the mean MFI per marker within each group. (B) Pre-treatment (PRE) samples. (C) Post-treatment (POST) samples. (D) POST samples from non-pCR patients. (E) POST samples from pCR patients.

Additional file 5

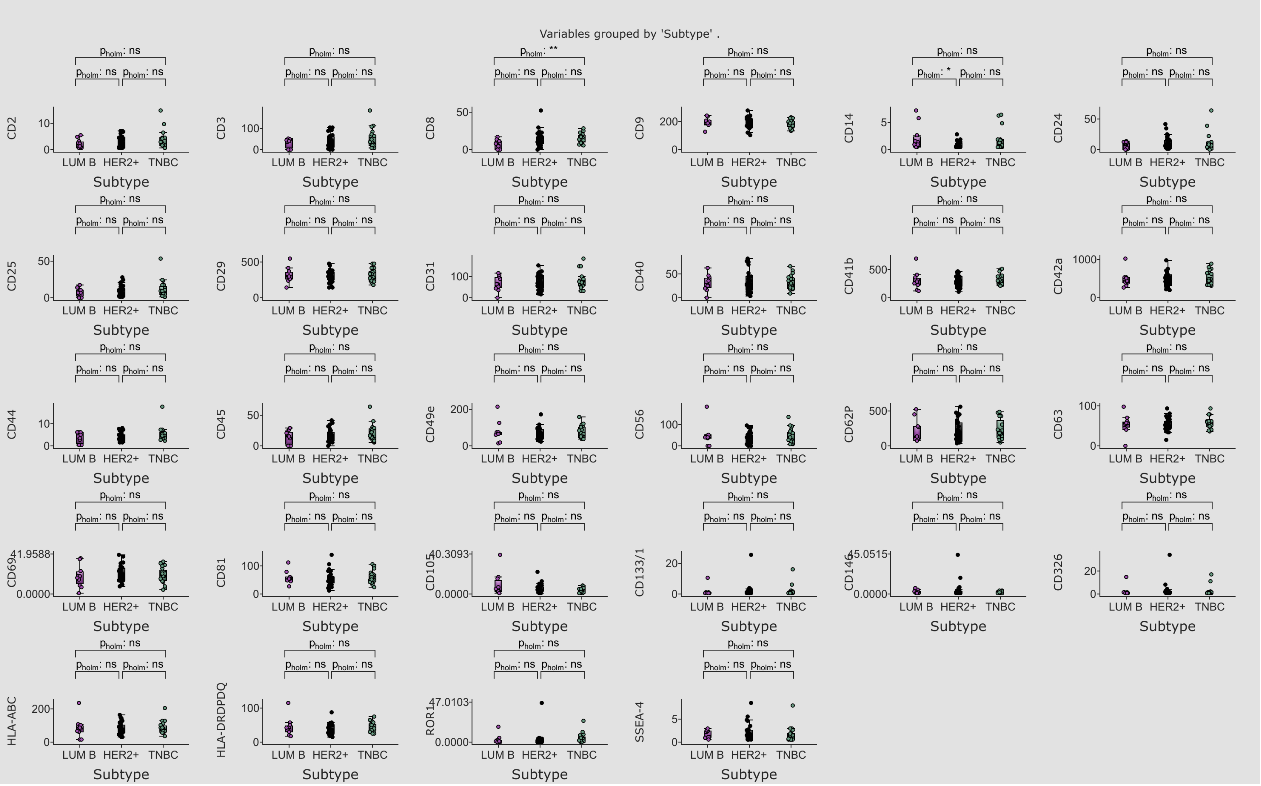

Additional file 5. EV surface marker expression in PRE samples across breast cancer subtypes.

Surface marker expression in EVs isolated from plasma collected before neoadjuvant systemic treatment (NST), grouped by breast cancer subtype: luminal B (LUMB), HER2-positive (HER2+), and triple-negative breast cancer (TNBC). Data represent CD9/CD63/CD81-normalized median fluorescence intensity (MFI) for each marker retained after threshold filtering. Bar graphs show mean values with standard deviation (SD).

Statistical comparisons were performed using one-way ANOVA with Holm correction for multiple comparisons. *p < 0.05, ** p < 0.01, “ns” = not significant.

Additional file 6

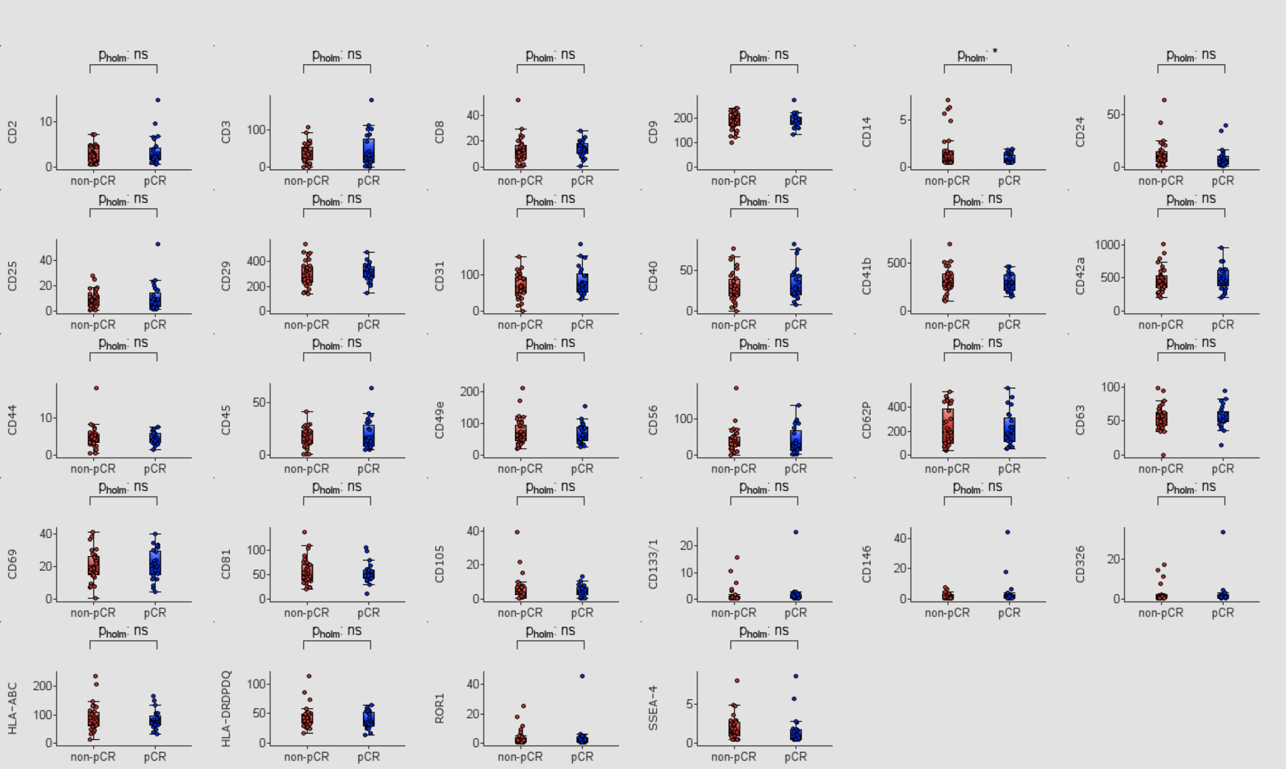

Additional file 6. Pre-treatment characteristics across pathological complete responders (pCR) and patients with residual disease (non-pCR) after neoadjuvant treatment.

Box plots showing CD9/CD63/CD81-normalized median fluorescence intensity (MFI, APC channel) for the 28 MACSPlex markers retained after threshold filtering (MFI > 0.5 in at least 50% of samples). Marker expression is grouped by treatment response: pathological complete response (pCR) vs. non-pCR.

Statistical comparisons were performed using unpaired t-tests with Holm correction for multiple testing. A p-value < 0.05 was considered statistically significant and is indicated by *; non-significant results are labeled “ns”. Box plots show the median, interquartile range (IQR), and full range; individual patient values are plotted as dots.

Additional file 7

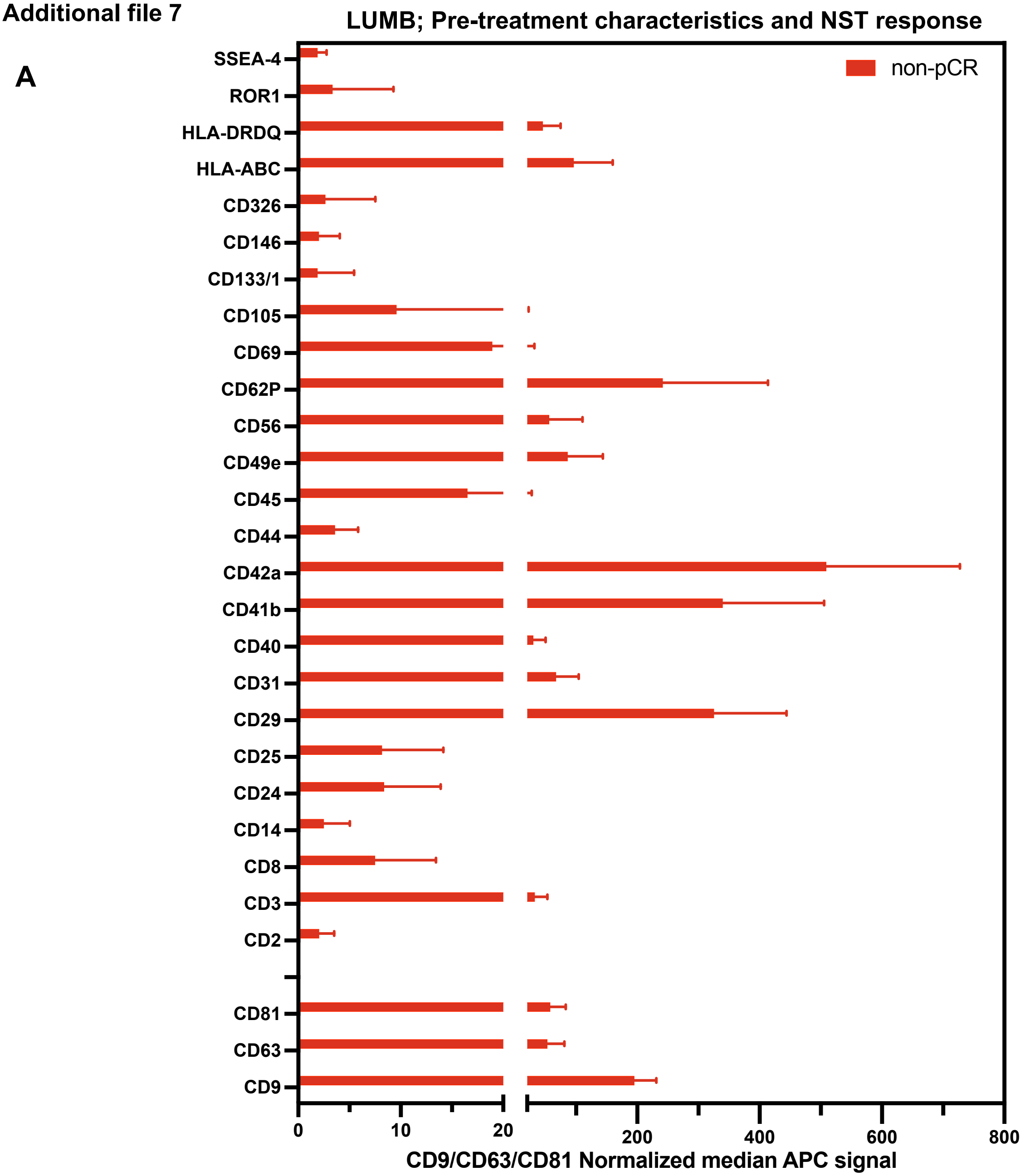

Additional file 7

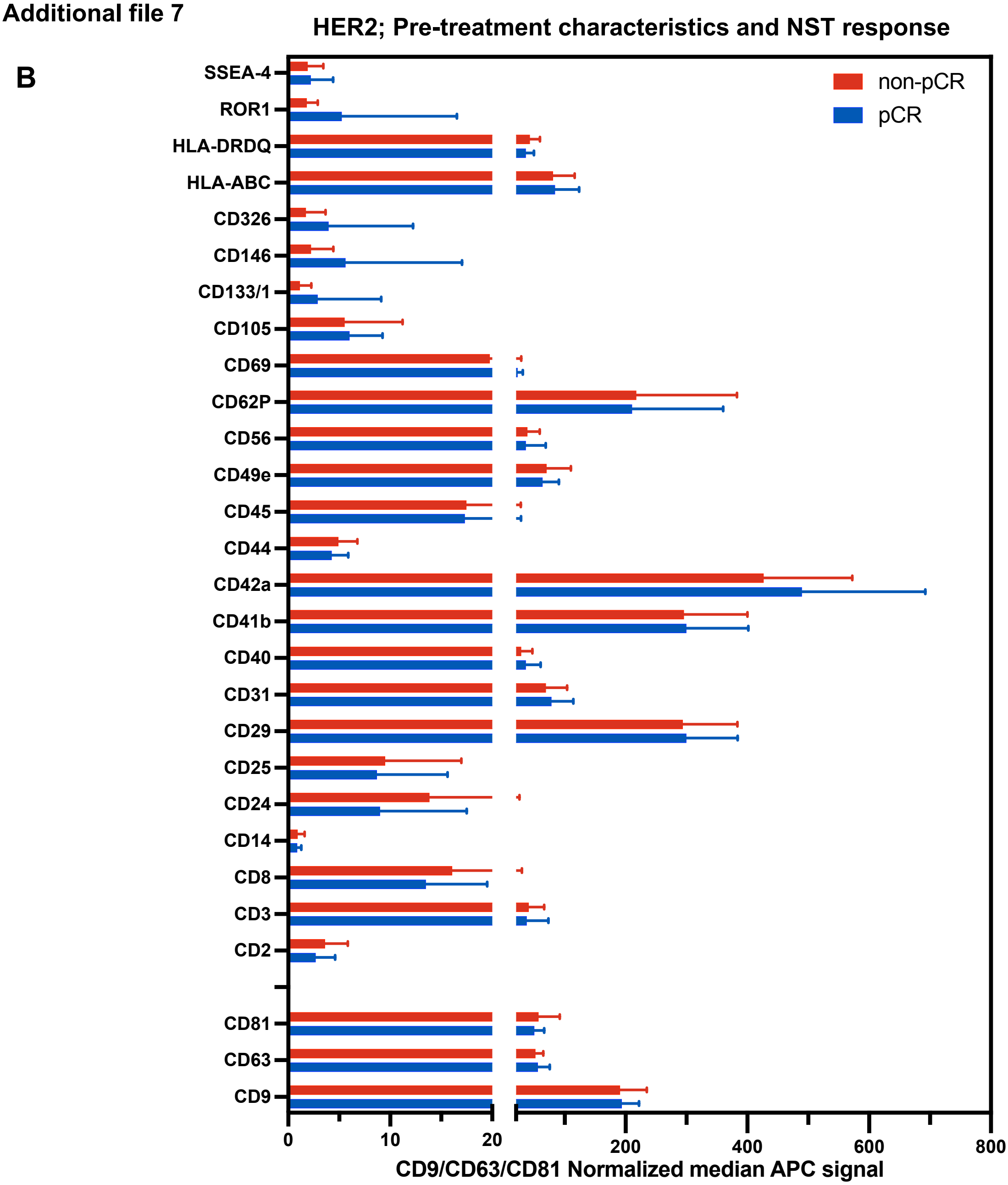

Additional file 7

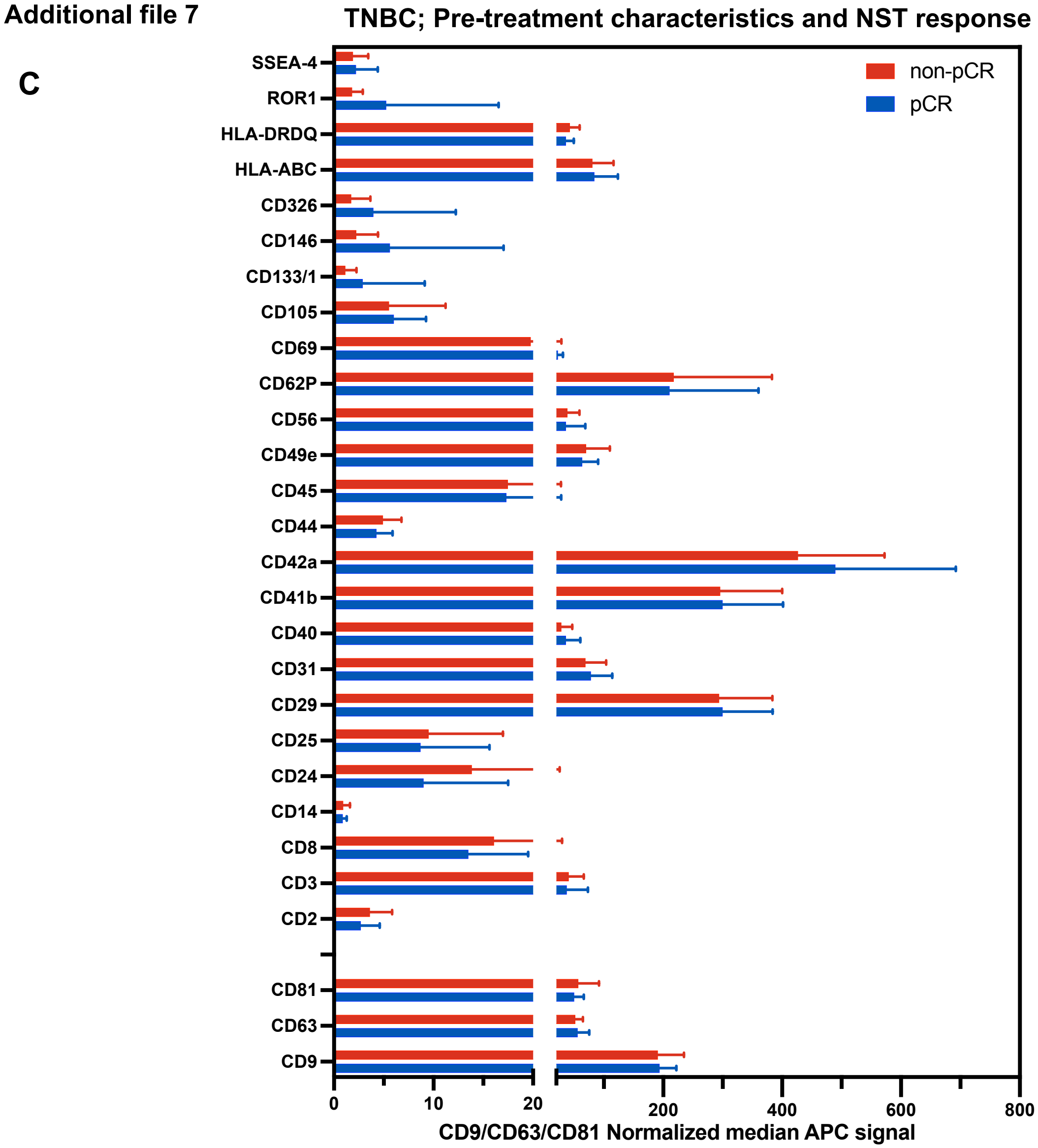

Additional file 7. Pre-treatment characteristics across breast cancer subtypes according to pathological response (pCR vs. non-pCR) after neoadjuvant treatment.

Surface marker expression in plasma-derived EVs collected before neoadjuvant systemic treatment (PRE), analyzed using the MACSPlex assay. Values represent CD9/CD63/CD81-normalized median fluorescence intensity (MFI, APC channel) for the 28 markers retained after threshold filtering (MFI > 0.5 in at least 50% of samples). Marker expression is grouped by treatment response within breast cancer subtypes: (A) Luminal B-like (LUMB), (B) HER2-positive (HER2+), and (C) triple-negative breast cancer (TNBC). Note that all EV samples were collected before treatment, whereas response was evaluated after completion of NST. Bars represent mean values with standard deviation (SD).

Statistical comparisons were performed using unpaired t-tests with Holm correction for multiple testing. A p-value < 0.05 was considered statistically significant and is indicated by *; non-significant results are labeled as “ns”.

Additional file 8

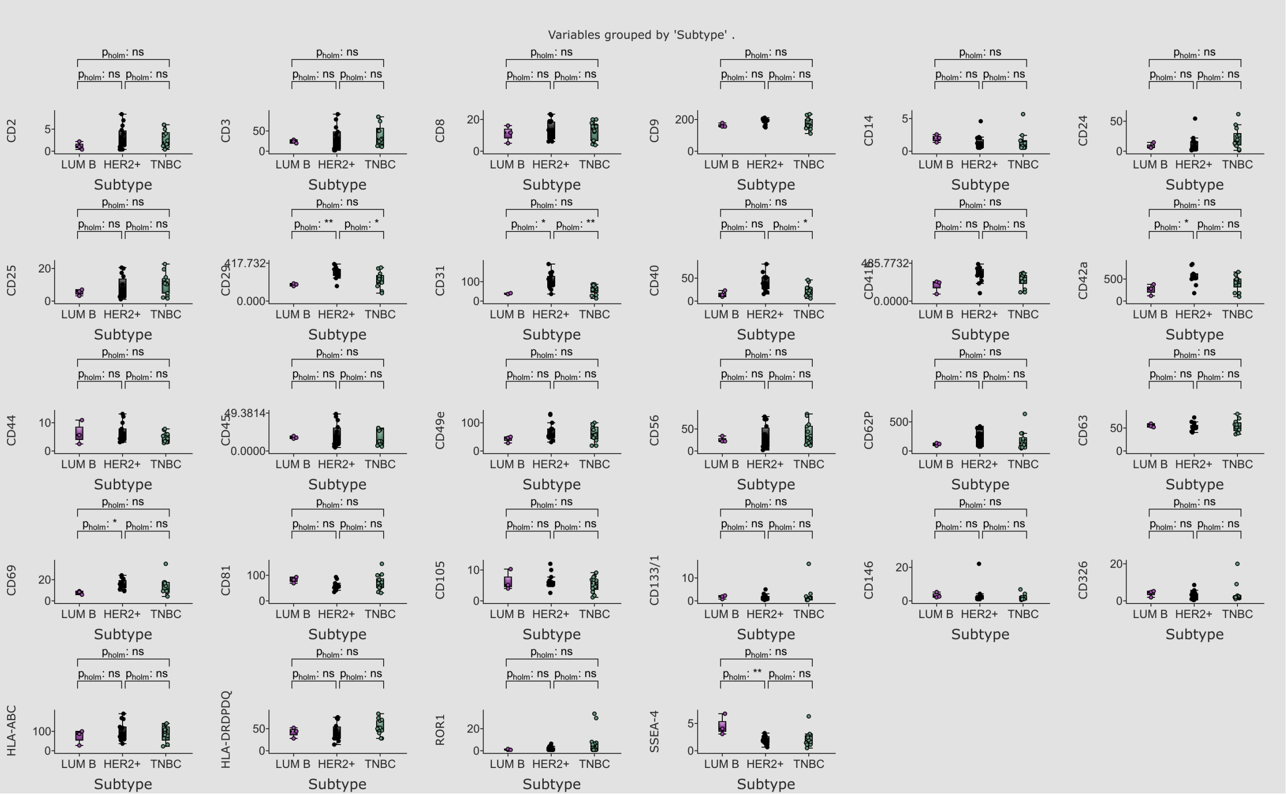

Additional file 8. EV surface marker expression in samples collected after neoadjuvant systemic treatment (NST), stratified by breast cancer subtype.

Box plots show CD9/CD63/CD81-normalized median fluorescence intensity (MFI, APC channel) for the 28 MACSPlex markers retained after threshold filtering (MFI > 0.5 in ≥50% of samples). Marker expression is grouped by breast cancer subtype: Luminal B-like (LUMB), HER2-positive (HER2+), and triple-negative breast cancer (TNBC).

Statistical comparisons were performed using one-way ANOVA with Holm correction for multiple testing. A p-value < 0.05 was considered statistically significant and is indicated by *; non-significant comparisons are labeled “ns”. Box plots indicate the median, interquartile range (IQR), full range, and individual values.

Additional file 9

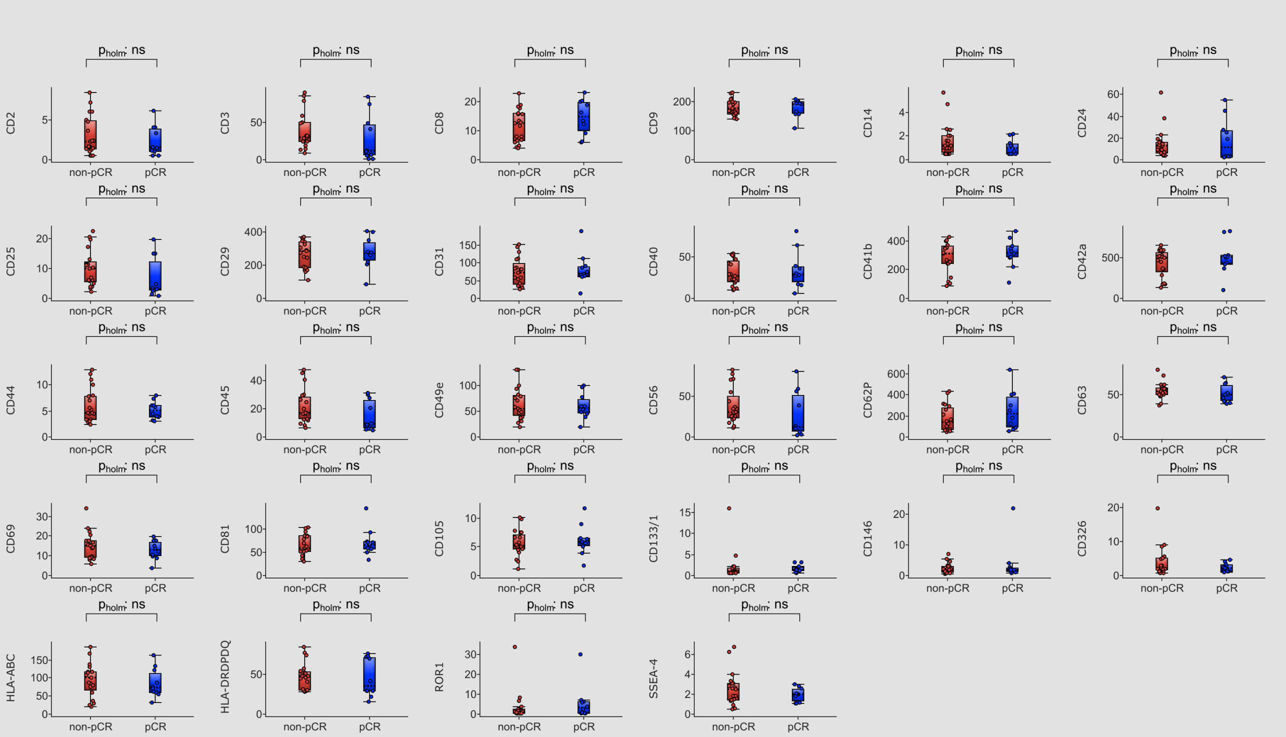

Additional file 9. EV surface marker expression in samples collected after neoadjuvant systemic treatment (NST), stratified by treatment response.

Box plots show CD9/CD63/CD81-normalized median fluorescence intensity (MFI, APC channel) for the 28 MACSPlex markers retained after threshold filtering (MFI > 0.5 in ≥50% of samples). Marker expression is grouped by pathological complete response (pCR) versus non-pCR status.

Statistical comparisons were performed using unpaired t-tests with Holm correction for multiple testing. A p-value < 0.05 was considered statistically significant and is indicated by *; non-significant comparisons are labeled "ns". Box plots indicate the median, interquartile range (IQR), full range, and individual values.

Additional file 10

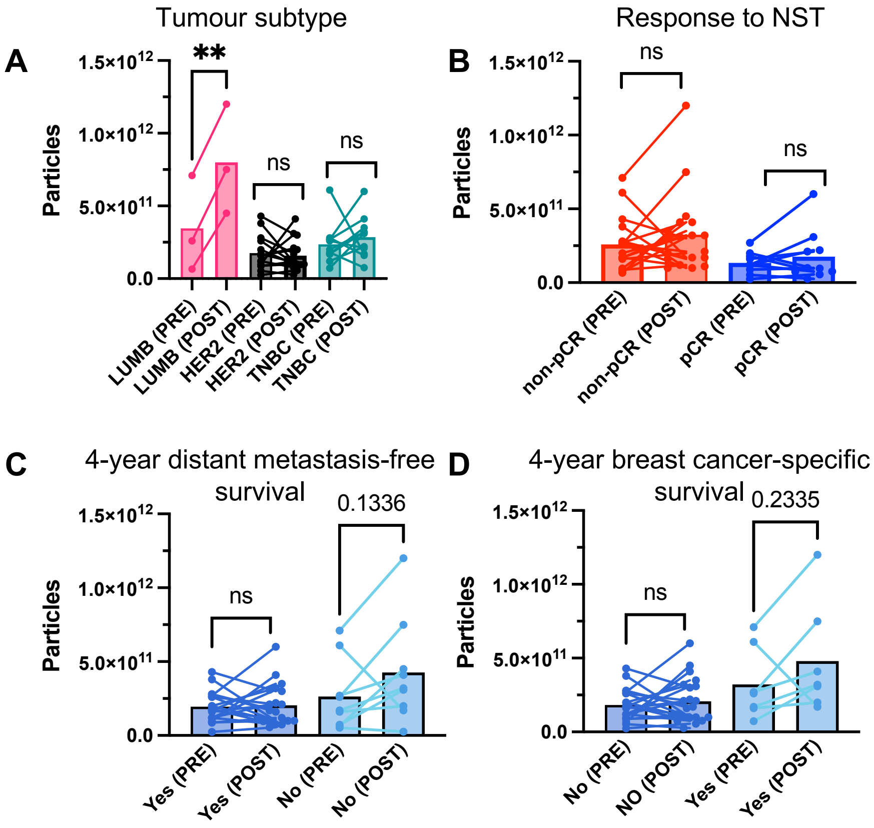

Additional file 10. Paired EV particle concentration before and after NST across subtypes and clinical outcome groups.

EV particle concentration (measured by NTA) in paired PRE and POST plasma samples, shown for individual patients. Grouped by (A) breast cancer subtype: luminal B (LUMB), HER2-positive (HER2+), and TNBC, (B) pathological response: pCR vs. non-pCR, (C) distant metastasis and (D) breast cancer-specific mortality within four years.

Statistical comparisons were performed using paired t-tests within each group. A p-value < 0.05 was considered statistically significant and is indicated by *; “ns” denotes non-significant comparisons. Plots show individual patient changes from PRE to POST with group means and standard deviation (SD).

Additional file 11

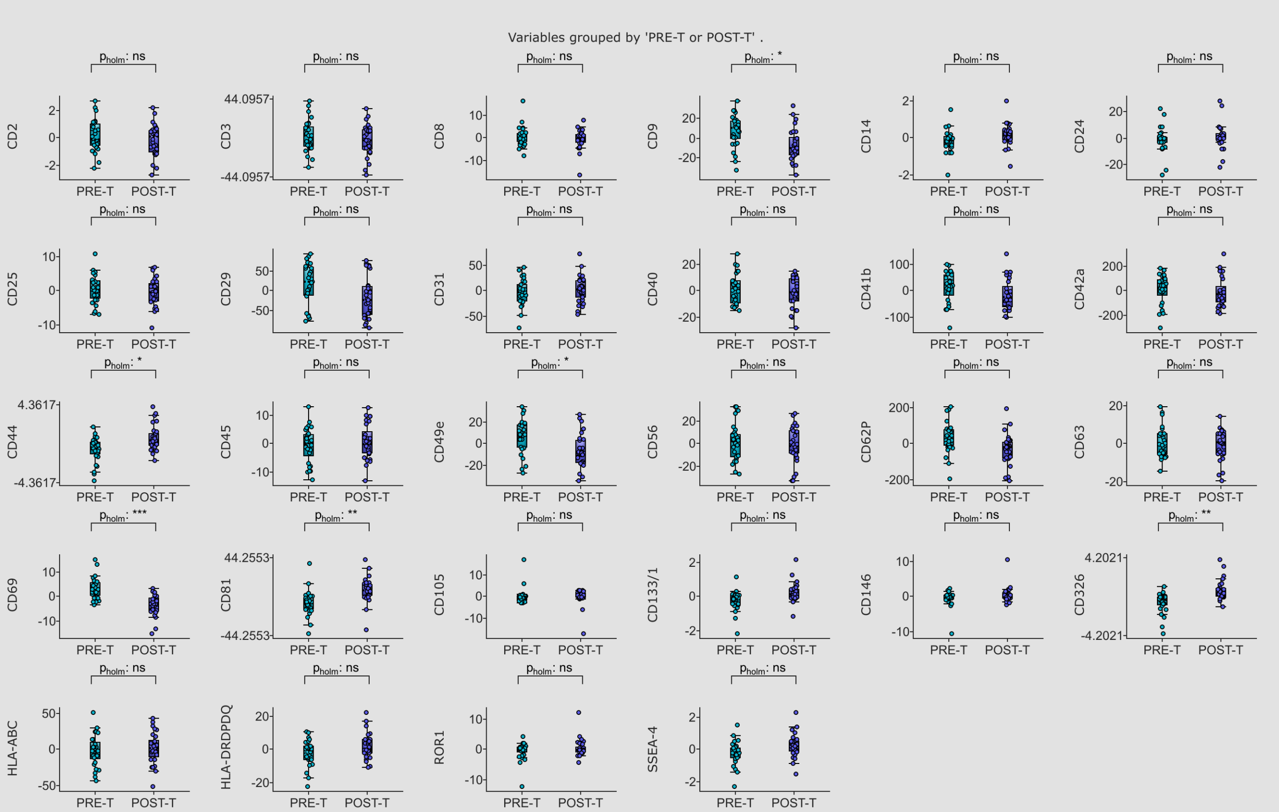

Additional file 11. Paired EV surface marker expression before and after neoadjuvant systemic treatment (NST).

CD9/CD63/CD81-normalized median fluorescence intensity (MFI, APC channel) for 28 MACSPlex markers, measured in paired plasma samples collected before (PRE) and after (POST) NST. Box plots show the median, interquartile range (IQR), and full range; individual patient values are plotted as paired dots.

Statistical comparisons were performed using paired t-tests with Holm correction for multiple testing. Statistical significance is indicated as * $p < 0.05$; ** $p < 0.01$; ns = not significant.

Additional file 12

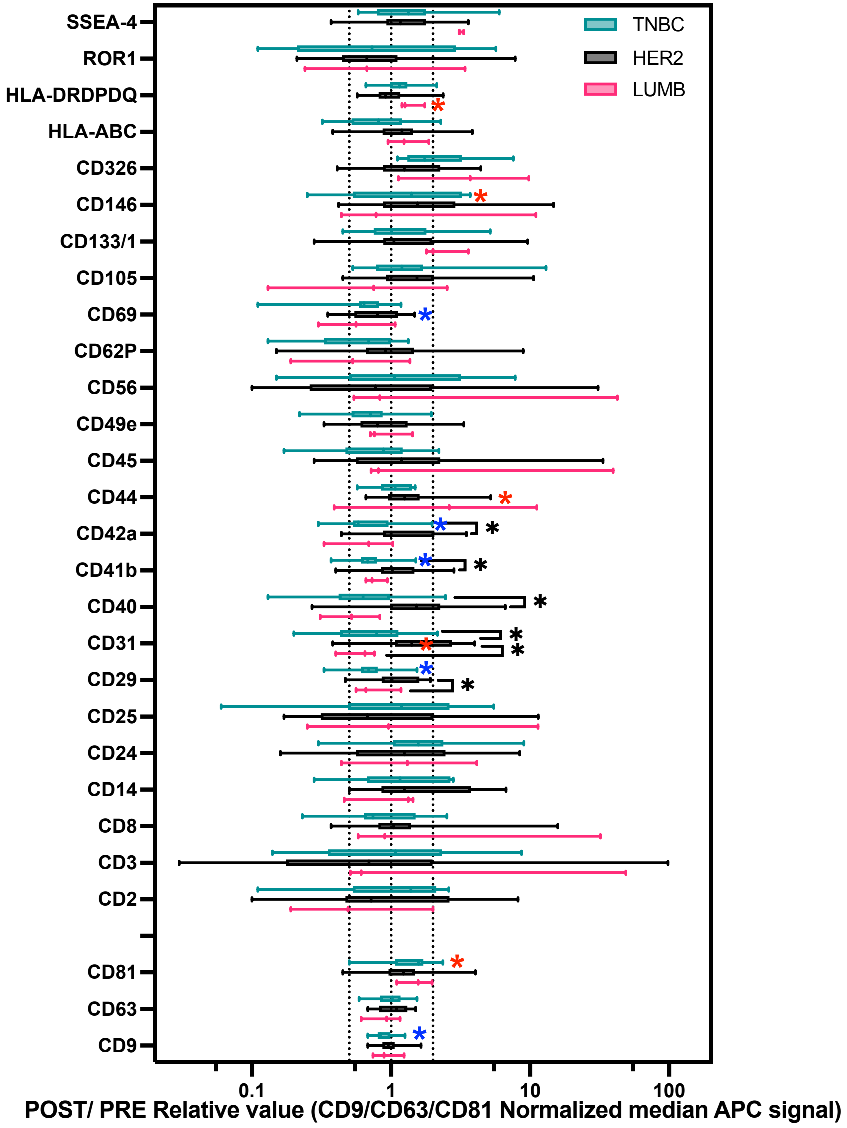

Additional file 12. EV surface marker expression changes in response to neoadjuvant systemic treatment (NST), stratified by breast cancer subtype.

Surface marker expression in plasma-derived EVs before and after NST, assessed using MACSPlex analysis. Data are presented as the POST/PRE ratio of CD9/CD63/CD81-normalized APC signal, representing the individual fold change. For each marker, fold changes are shown across breast cancer subtypes: Luminal B-like (LUMB), HER2-positive (HER2+), and triple-negative breast cancer (TNBC). Box plots display the median, interquartile range, and full range on a log scale; values >1 indicate increased expression post-treatment, and values <1 indicate decreased expression.

Statistical comparisons were performed using paired t-tests with Holm correction. Colored asterisks indicate significant within-group changes relative to baseline (PRE): red asterisks indicate significant upregulation post-treatment, and blue asterisks indicate significant downregulation. Significance level: *p < 0.05, **p < 0.01.
